# Mixed Halide Isothiocyanate Tin(II) Compounds, SnHal(NCS): Signs of Tetrel Bonds as Bifurcated Extensions of Long-Range Asymmetric 3c-4e Bonds

**DOI:** 10.3390/molecules30132700

**Published:** 2025-06-23

**Authors:** Hans Reuter

**Affiliations:** Chemistry, Department of Biology/Chemistry, Osnabrück University, Barbarastr. 7, D 49069 Osnabrück, Germany; hreuter@uos.de

**Keywords:** tin(II), coordination spheres, coordination geometries, interpenetration indices, bond valences, 3c-4e-bonds, tetrel bonds, σ-hole, bifurcation, structure–bonding relationships

## Abstract

As part of a systematic study on the structures of the mixed halide isothiocyanates, Sn^II^Hal(NCS), their single crystals were grown and structurally characterized. For Hal = F (**1**), the SnClF structure type was confirmed, while with Hal = Cl (**2**), Br (**3**), and I (**4**), there are three isostructural compounds of a new structure type, and for Hal = Cl (**5**), there is a second modification of a third structure type. These structure types have been described with respect to the composition and coordination geometry of the first, second, and van der Waals crust coordination spheres and their dependence on the halogen size and thiocyanate binding modes. With respect to the first coordination spheres, all three structure types constitute one-dimensional coordination polymers. In **1**, “ladder”-type double chains result from μ_3_-bridging fluorine atoms, and in **2**–**4**, single-chains built up from μ_2_-halogen atoms are pairwise “zipper”-like interconnected via *κ*^2^*NS*-bridging NCS ligands, which manage the halogen-linked chain assembly in the double chains of **5**. Based on the octet rule, short atom distances are interpreted in terms of 2c-2e and various (symmetrical, quasi-symmetrical, and asymmetrical) kinds of 3c-4e bonds. Weak contacts, the topology of which suggests the extension of the latter bonding concept, are identified as electron-deficient, bifurcated tetrel bonds.

## 1. Introduction

With its valence electron configuration, (5s)^2^(5p)^2^, tin is predestined to form compounds in the oxidation states +IV and +II [1,2,3]. In SnX_4_ compounds with monovalent atoms, or assemblies, X, the tetravalent tin atom is usually tetrahedrally coordinated, which is due to its sp^3^ hybridization and classical two-centre, two-electron, 2c-2e bonds. Typical examples are found in the case of the molecular *tetrahalides* SnHal_4_ with Hal = Cl [4], Br, and I [5], while the tin atom in the *tetrafluoride* SnF_4_ is hexacoordinated due to fluorine bridges [6]. Molecular tin(IV) compounds are characterized by the fact that the atom distances d(Sn-X) have a narrow bandwidth, so it is possible to calculate them via the addition of the normal covalent radii of Sn and X.

A completely different picture emerges for divalent tin. In SnX_2_ compounds, the tin atom actually only has six valence electrons, which means that they are nominally electron-deficient compounds, as they are two electrons short of the electron octet of a p-block element. In the solid state, the tin atom in these compounds usually achieves this stable electron configuration via interaction with a free electron pair of an already bonded substituent X of a second tin atom, resulting in polymeric structures. As the bond formation takes place exclusively via the three orthogonal p-orbitals, a one-sided, hemi-directed [7] ligand distribution around the tin atom results in contrast to the spherical, holo-directed [7] ligand distribution in the case of SnX_4_.

However, this one-sided ligand distribution proves to be unfavourable in most cases, which is why, in the solid-state structure of many tin(II) compounds, further but weaker tin–ligand contacts, Sn~X, occur. As a result, tin(II) compounds exhibit much broader bond length distributions in the solid state than in comparable tin(IV) compounds, often ranging from the covalent boundary B_cov_ = r_cov_(Sn) + r_cov_(X) up to the van der Waals boundary B_vdW_ = r_vdW_(Sn) + r_vdW_(X). These additional contacts of the hyper-coordinated tin(II) atom can no longer be explained on the basis of 2e-2c bonds alone because tin—like all other p-block elements—must obey the octet rule [8]. Instead, additional 3c-4e bonds, according to the Pimentel–Rundle concept [9,10,11], must be taken into account in order to avoid hyper-valency and violation of the octet rule.

In the context of structure–bonding relationships, two benchmarks of tin(II) compounds are of special interest: first, the atom distance d(Sn-X) of a classical 2c-2e bond, and second, the atom distance d(Sn-X) in the case of two equidistant, *trans*-positioned atoms, X, in a three-centre X-Sn-X arrangement, because this bonding situation (equidistant bond lengths, *trans* position, and seesaw coordination) provides strong evidence for symmetrical 3c-4e bonds according to the Russel–Pimentel concept. Prototypes of the first benchmark are found in electron-precise tin(II) species with a trigonal pyramidal, *tpy* “SnX_3_” conformation, which is found in isolated or “naked” “SnX_3_” species, while prototypes for the second are found especially in hyper-coordinated tin(II) species, “SnX_4_”, with one pair of *trans*-positioned, equidistant X atoms in a quasi-linear arrangement.

In the case of X = Hal, data for a great number of [SnHal_4_]^2-^ species are nowadays available for Hal = Cl [12,13,14,15], Br [13,15,16,17,18,19], and I [15,20,21,22], all with terminal halogen atoms. Crystal structures with higher coordinated halogen atoms are currently unknown for these ligands. For Hal = F, the situation is reversed: no isolated [SnF_4_]^2-^ ions with terminal fluorine atoms are known, except for seesaw “SnF_4_” units with μ_2_- and μ_3_-bridging fluorine atoms. An example of the former is found in the coordination compound 3SnF_2_ · 2phen · 2MeOH [23], **a**, while examples of the latter can be found in the inorganic compounds SnF(NCS) [24], **b**, and SnClF [25], **c**, respectively. In these compounds, the Sn-F atom distances and F-Sn-F bond angles under consideration are 2.292(2)/156.01(7)° (**a**), 2.391(4)/131.8(4)° (**b**), and 2.398(6)/130.0(6)° (**c**). The influence of the fluorine coordination mode on both values [bond length increasing, bond angle decreasing with *N* of μ*_N_*] is obvious. The very small bond angle for μ_3_-X looks very unusual but is confirmed by a similar value [130.0(1)°] observed for X = μ_3_-OH in the compound Sn_6_O_4_(OH)_4_ [26].

In searching for tin(II) compounds in which the structural requirements of μ_3_-coordinated halogen atoms in a *trans* Hal-Sn-Hal arrangement are fulfilled for chlorine, bromine, and iodine, which would allow for the determination of their benchmark in question, we became aware of a note in [27] that all other representatives of the mixed halide isothiocyanates, SnHal(NCS), should be isostructural with the fluorine compound, but this statement has not been confirmed. We, therefore, decided to reinvestigate the crystal structures of these compounds in order to determine the atomic parameters. For the preparation and crystal growth of SnF(NCS), **1**, SnCl(NCS), **2**, SnBr(NCS), **3**, and SnI(NCS), **4**, we used the method previously described [28]. By use of this method, the detailed analysis of the crystals formed leads to the discovery of a second β-modification of SnCl(NCS), **5**, the structure of which proved to be completely different from **2**.

In the course of our investigations, it turned out that the original assumption was incorrect. The three orthorhombic crystallizing compounds **2**–**4** are isostructural among each other but differ from the structure of the fluorine compound. Nevertheless, the measurements reveal structure–bonding relationships that offer new insights into the coordination chemistry of the divalent tin atom as they prove (i) for the first time the influence of the different halogen atoms, their size, and their electronegativity on the interatomic distances and angles in the coordination sphere of tin(II) atoms, as they show (ii) the ambivalence of the thiocyanate ligand which can bind—as an expression of its great coordination ability [29,30]—to the tin atom unidentate via either the nitrogen or the sulphur atom or bidentate via both, and as they reveal (iii) that the occurring weak, so-called non-covalent interactions or tetrel bonds can be interpreted topologically as a pendant to similar hydrogen bonds as extensions of bifurcated, asymmetric 3c-4e bonds.

## 2. Results

The results of the crystal structure determinations (Table 1) at low temperatures confirm previous data with respect to the unit cell parameters and the orthorhombic space group *Pnma* of **1**–**4** and extend the existence range of these structures up to 100(2) K. The first indications that these compounds are probably not isostructural—as originally assumed—were obtained by means of comparison of the unit cell volumes and lattice parameters (Figures S1 and S2). These values correlate very well with the van der Waals radii [31] of the halogen atoms in the case of Hal = Cl, Br, and I but less well when the values of Hal = F are also taken into account. With respect to the ß-modification of SnCl(NCS), which has a lower density [3.283 g/cm^3^] than the orthorhombic α-modification [3.350 g/cm^3^], a completely different structure was expected, as **5** crystallizes in the triclinic space group *P*$\bar{1}.$

## 3. Discussion

Based on the explanations given at the beginning, it seems appropriate to interpret atom distances and coordination spheres in terms of 2c-2e and 3c-4e bonds. On this basis, it makes sense to classify the coordination spheres of divalent tin atoms as first, second, and van der Waals crust coordination spheres, whereby the first comprises all ligand atoms with whose electrons the tin atom reaches an electron octet, while it apparently exceeds this with the electrons from the additional ligand atoms of the second and van der Waals crust coordination spheres. The fundamental difference between the additional ligands of the second and van der Waals crust coordination spheres results from the atom distances, which are shorter in the former case than in the latter, where they reach values comparable to the sum of the van der Waals radii of tin and the ligand atom.

### 3.1. First Coordination Spheres

#### 3.1.1. SnF(NCS), **1**

**Figure 1:**
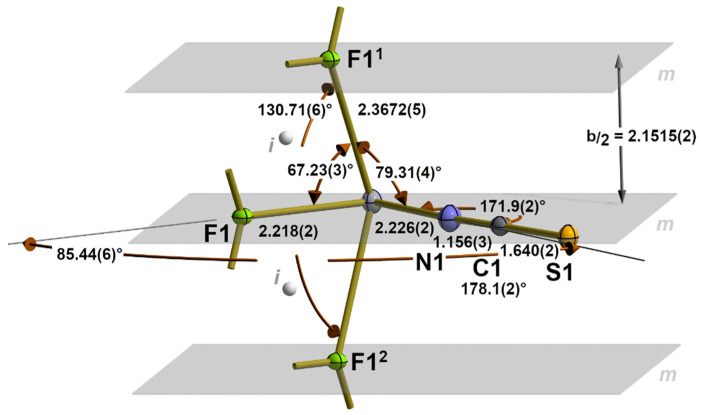
First seesaw 3_1_-s coordination sphere of the tin atom in **1** with atom labelling and main bond lengths [Å], angles, and symmetry elements (*m* = mirror plane depicted in grey; *i* = centre of symmetry depicted as grey sphere). Symmetry transformations used to generate equivalent atoms: (^1^) −x, 1/2 + y, 1 − z; (^2^) −x, −1/2 + y, 1 − z.

The first coordination sphere of the tin atom in **1** is composed of four atoms in a seesaw-shaped “SnF_3_N” arrangement since, in addition to a nitrogen and fluorine atom in equatorial (*eq*) positions, two further fluorine atoms are exactly equidistant from the tin atom in axial (*ax*) positions (Figure 1). These equal distances [d(Sn-F)*_ax_*= 2.3672(5) Å] result from a crystallographic mirror plane on which the tin atom and the two equatorial atoms lie.

This fourfold coordination sphere, therefore, represents the prototype of a hyper-coordinated tin atom with a symmetrical 3c-4e bond between the two axial fluorine atoms, a bonding and coordination mode that we previously denoted as 3_1_-s [28], wherein the number “3” indicates the number of electron pairs necessary for the bivalent tin atom to achieve the electron octet, the index “1” represents the number of 3c–4e bonds present, and the letter “s” stands for the symmetrical type of this bond. Adding the number of electron pairs (three) and the index (one) together provides the total number of bonded atoms (X = 4) for this tin coordination, and together with the non-bonding electron pair Y, the expression (SnX_4_Y) and the geometry of this coordination according to the VSEPR concept are obtained [32].

With respect to the benchmark of the symmetrical 3c-4e bond, the value determined in the present work is not only of higher precision but also somewhat shorter [2.3672(5) Å] than the value [2.398(6) Å] resulting from the room-temperature measurement [24], while the bond angle [130.71(6)°] is somewhat larger [130.0(6)°]. In the isostructural compound SnClF [25], also measured at room temperature, both values [2.391(4) Å/131.8(4)°] are in the same order of magnitude.

Although the tin atom exhibits point group symmetry *C_m_*, the coordination geometry is characterized by very different bond angles ranging from 85.44(6)° between the equatorial to 130.71(6)° between the axial atoms. In comparison, the bond angles between the equatorial and axial ligands are very small, at 67.23(3)° in the case of F*_eq_* and 79.34(4)° in the case of N*_eq_*_._

Important reference values for the Sn-N and Sn-S atom distances of the NCS ligand are found in the crystal structure of Sn(NCS)_2_, which represents, in a solid state, a one-dimensional coordination polymer [33]. In this compound, the first coordination sphere of the tin atom consists of one terminal *κ*^1^*N* [d(Sn-N) = 2.198(2) Å] and one bridging *κ*^2^*NS* [d(Sn-N) = 2.284(2) Å, d(Sn-S) = 2.8209(5) Å] thiocyanate group, resulting in a *tpy* “SnN_2_S” coordination geometry with the tin atom at the apex and the nitrogen and sulphur atoms in the basal plane. The value of the Sn-N atom distance [d(Sn-N*_eq_*) = 2.226(2) Å] observed in the crystal structure of **1** lies in between both. Meanwhile, much shorter Sn-N bonds [2.172(2) Å] are observed in the case of the 3_1_-s coordinated tin atom of Sn(NCS)_2_ · 2H_2_O · 2(18-crown-6) [34].

The outstanding feature of **1** represents the fluorine atom, as it simultaneously bridges three tin atoms, being located once in the equatorial and twice in the axial position of the same tin atom. As a result, Sn-F distances are relatively long, even in the case of its equatorial position [d(Sn-F)*_eq_* = 2.218(2) Å], for which a 2c-2e bond must be assumed. The benchmark for the length of this kind of tin–fluorine bonds comes from isolated, monomeric, trigonal–pyramidal [SnF_3_]^−^ ions, where bond lengths in the range of 1.992(2)–2.002(1) Å [mean value: 2.001(2) Å] [23] are found. Even shorter [1.991(1) Å] Sn-F distances have previously been observed in the first coordination sphere of a tin atom in the phenanthroline coordination compounds 3SnF_2_ · phen [28] and 3SnF_2_ · 2phen · 2MeOH [23]. In both compounds, the relevant fluorine atoms adopt equatorial positions in a seesaw “SnF_4_” coordination, as in **1**, but they do not bridge two or three tin atoms, nor are their distances to the tin atom influenced by an atom of the second coordination sphere in the *trans* position. By way of comparison, it should be noted that the sum of the covalent radii [35] of tin and fluorine rises to 1.96 Å.

**Figure 2:**
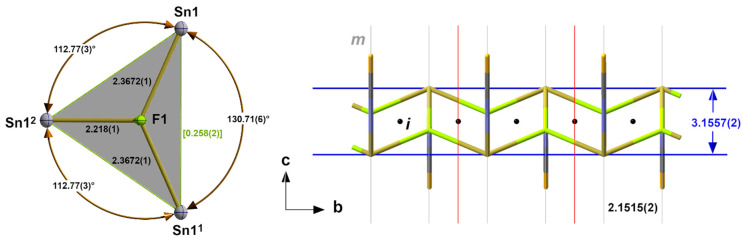
Ball-and-stick model (left) of the almost trigonal–planar coordinated fluorine atom in **1** with the bond lengths [Å], angles, and distance [Å] of the fluorine atom above the basal plane in square brackets (green); stick model (right) of the “ladder”-like arrangement of the tin and fluorine atoms in **1** with distances [Å] between “rails” and “rungs” and the position of crystallographic mirror planes *m* and inversion centres *i*; symmetry transformations used to generate equivalent atoms: (^1^) x, −1 + y, z; (^2^) −x, −1/2 + y, 1 − z.

The μ_3_-fluorine atom is almost trigonal–planar coordinated. The sum of the bond angles rises to 356.25° with the fluorine atom 0.258(2) Å above the plane of the three tin atoms (Figure 2).

In summary, the interaction of the tin and fluorine atoms results in the formation of double chains with a “ladder”-like arrangement of their building units characterized by a distance of 3.1557(2) Å between the “rails” (=Sn-Atoms) and a distance of b/_2_ = 2.1515(2) Å between the “rungs” (=b/2). The compact μ_3_-coordination of the fluorine atom prevents it from making further contact with other tin atoms in the neighbouring “ladder”. These contacts are exclusively restricted to the sulphur atoms of the thiocyanate ligand and are discussed below.

#### 3.1.2. α-SnCl(NCS), **2**, SnBr(NCS), **3**, SnI(NCS), **4**

**Figure 3:**
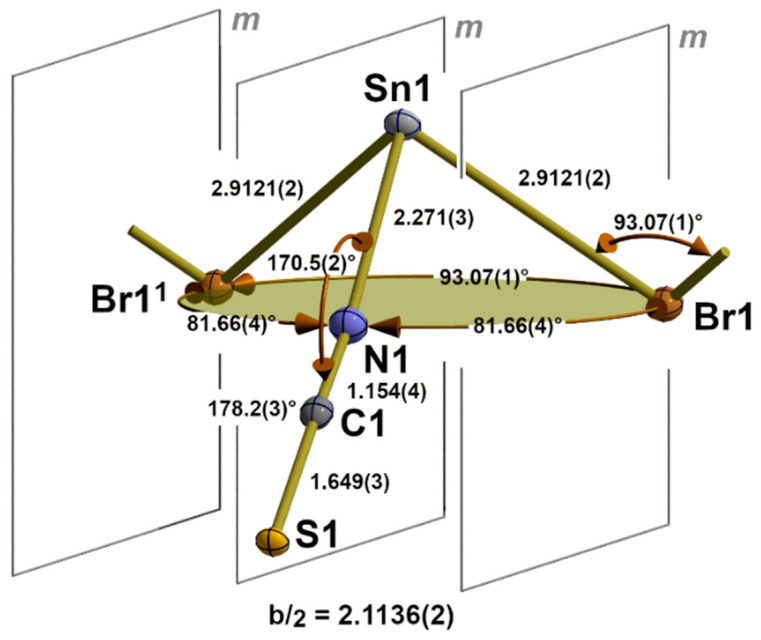
Details of the one-dimensional coordination polymer in the crystal structure of **3** showing the first, *tpy* “SnBr_2/2_(NCS)” coordination sphere (base of the pyramid in green) of the tin atom and position of the crystallographic mirror planes, *m*; selected bond lengths [Å] and angles; symmetry transformations used to generate equivalent atoms: (^1^) x, y + 1, z.

Deviating from the expected 3_1_-s coordination, the first coordination sphere of the tin atoms in this family of isostructural compounds exhibits a *tpy* 3_0_-coordination mode (index 0 = no 3c-4e bond) consisting of two halogen atoms and one nitrogen atom of an NCS group (Figure 3) in the basal plane and the tin atom at the apex of a trigonal pyramid. As all atoms are on crystallographic mirror planes, the halogen atoms act as bridges between two tin atoms, resulting in a chain-like arrangement of the trigonal pyramids. Bond lengths and angles of these compounds are summarized in (Table 2).

These one-dimensional coordination polymers with μ_2_-bridging halogen atoms and terminal isothiocyanate ligands are very similar to those found in the thermodynamical stable modifications of the corresponding *dihalides*. Due to the lack of comparable high-quality, low-temperature single-crystal data, we draw on data presented at the 23rd Annual Conference of the German Crystallographic Society [36]. In the *dihalides*, the distances between the tin atom and the bridging halogen atoms are only insignificantly shorter in the case of SnCl_2_ [d(Sn-Cl) = 2.7629(4) Å] and SnBr_2_ [d(Sn-Br) = 2.919(4) Å], but in the case of SnI_2_ [d(I-Sn-I = 3.172(1) Å], they are significantly longer, due to the fact that the corresponding iodine atom not only bridges two tin atoms in the chain but also belongs to the coordination sphere of a second tin atom in an adjacent chain of octahedrally coordinated tin atoms.

Crystal structures of isolated 3_0_ “SnHal*_n_*(NCS)_3-*n*_” species are only known for *n* = 1 and Hal = Cl. The tin–chlorine distance of 2.564(1) Å for the terminal chlorine in the compound [NH_3_-(CH_2_)_2_-NH_3_][SnCl(NCS)_2_]_2_ [37] indicates, in comparison with the value [2.7754(3) Å] observed here, how strongly the bridging function influences the bond length. The two tin–nitrogen distances d(Sn-N) = 2.255(4), 2.281(5) Å are in the range observed here.

The internal structural parameters of the NCS ligands (Table 3) show no dependence in relation to the halogen atoms. This is also valid in the case of the (Sn-N-C) angles, which show a mean value of 170.7(2)°, comparable with the corresponding value in **1**. Atom distances between the nitrogen and carbon atoms correspond to N≡C triple bonds, while those between the carbon and sulphur atoms correspond to C-S single bonds, representing the most prominent bonding situation of this ligand [30].

**Figure 4:**
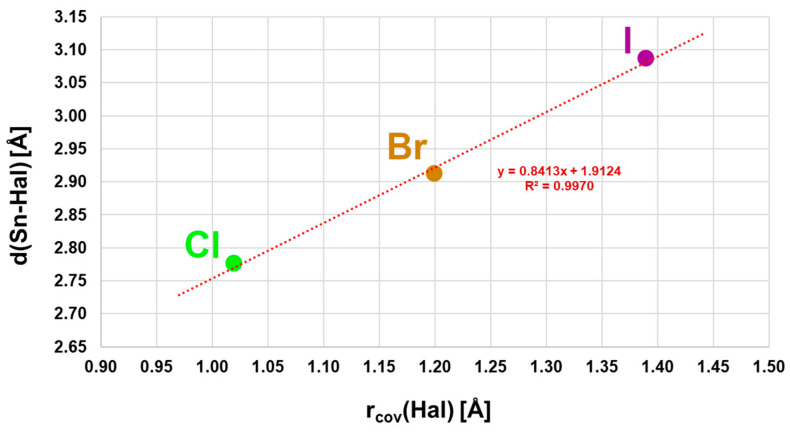
Correlation of the tin–halogen distances with the covalent radii of the halogen atoms in the α-SnCl(NCS) structure type.

With three different halogen atoms, the structure family of α-SnCl(NCS) provides an unusually deep insight into the influence of the halogen atom on the structural parameters. Thus, the intrachain tin–halogen bond lengths increase with increasing size of the halogen atoms from Hal = Cl to Hal = I (Figure 4), while the associated bond angles decrease (Figure S2), as do the distances ΔSn (Figure S3) of the tin atoms from the basal plane. Atom distances, bond angles, and ΔSn values are strongly correlated (R^2^ = 0.9970/0.9998/0.9970) with the covalent radii of the halogen atom.

#### 3.1.3. β-SnCl(NCS), **5**

**Figure 5:**
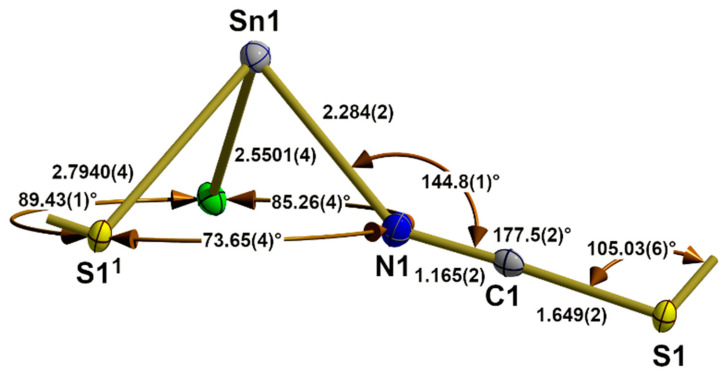
Asymmetric unit in the crystal structure of β-SnCl(NCS) with bond lengths [Å] and angles. Symmetry transformations used to generate equivalent atoms: (^1^) x, y − 1, z.

Similarly to the first coordination sphere of the tin atom in the α-SnCl(NCS) structure type, the tin atom of **5** exhibits a 3_0_-coordination mode but with one terminal chlorine atom and two bridging *κ*^2^*NS* thiocyanate ligands in the basal plane (Figure 5). With d(Sn-S) = 2.7904(4) Å, this kind of NCS bridge, therefore, provides an important value for the length of a 2c-2e tin–sulphur bond.

The end-to-end functionality of the thiocyanate ligand has a great influence on its internal structural parameters. While the C-S bond length and N-C-S bond angle are in the same magnitude as in the terminal thiocyanate ligands of **1** and the compounds of the α-SnCl(NCS) structure type, these values at the nitrogen atom undergo major changes: the N-C distance is significantly larger [1.165(2) to 1.155(5) Å], while the Sn-N-C bond angle [144.8(1)° to 170.8(3)°] is significantly smaller. The dihedral angle (Sn1-N1-S1-Sn1^2^) with Sn1^2^ in x,1 + y,z rises to −16.0(1)°.

In Sn(NCS)_2_ [33], the bridging thiocyanate ligand adopts the same coordination mode with similar external nitrogen [d(Sn-N) = 2.283(2), (Sn-N-C) = 146.5(2)°] and sulphur bonding parameters [d(S-Sn) = 2.8208(4), (C-S-Sn) = 104.2°]. With respect to the internal structural parameters, the carbon–sulphur distance [1.642(2) Å] and bond angle (N-C-S) [177.8(2)°] are of the same magnitude as in **5** [1.649(2) Å/177.5(2)°], but the N-C distance [1.161(3) Å] is somewhat shorter [1.165(2) Å]. The great similarity of the structural parameters (atom distances and angles) within the primary building units, as well as their linkage patterns (1D), is also reflected in the fact that both structures have a b-axis of similar length.

### 3.2. Second Coordination Spheres

The atoms that are added in the second coordination sphere are best identified by looking into the spatial arrangement of the one-dimensional chains built from the first coordination spheres described above. The associated interchain tin-to-ligand distances are longer, and the interactions are weaker than those of the first coordination spheres.

#### 3.2.1. SnF(NCS), **1**

**Figure 6:**
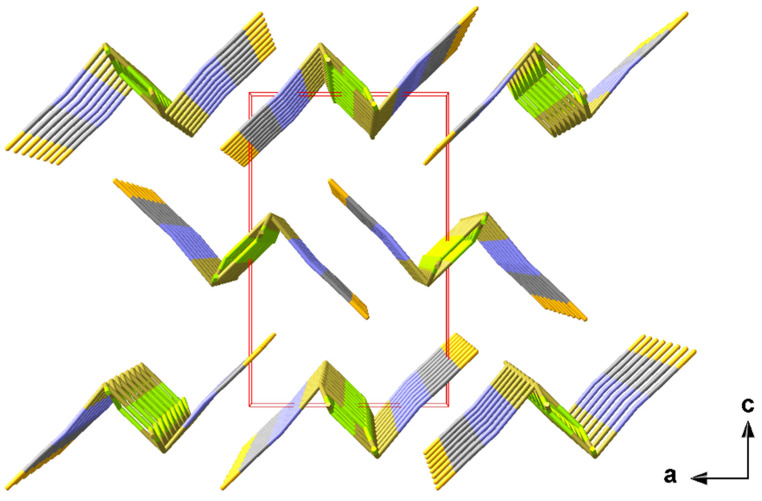
Perspective view of the crystal packing of **1** looking down the b-axis; stick model with unit cell presented in red.

The one-dimensional “ladders” resulting from the 3_1_-s coordination mode of the tin atoms are arranged in the direction of the crystallographic b-axis with an antiparallel orientation of the isothiocyanate ligands of two directly neighbouring “ladders” (Figure 6).

With 3.2860(1) and 3.5763(1) Å, the tin–sulphur distances between these “ladders” are too long to be interpreted as covalent interactions. Their impact on the coordination geometries at the tin and sulphur atoms is, therefore, described in detail in the section on weak interactions (2.3.1).

#### 3.2.2. α-SnCl(NCS), **2**, SnBr(NCS), **3**, SnI(NCS), **4**

**Figure 7:**
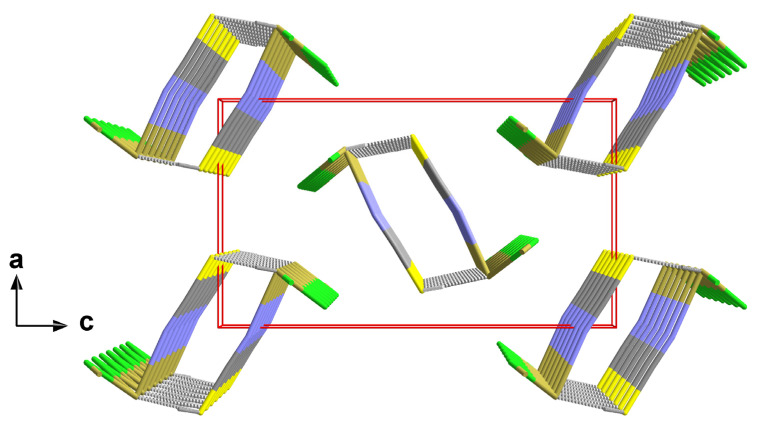
Perspective view of the crystal packing of **2**; stick model with unit cell presented in red looking down the b-axis; interchain tin–sulphur contacts between neighbouring chains are indicated by dashed sticks in grey.

In this structure type, the one-dimensional chains of the trigonal–pyramidal “Sn(μ_2_-Hal)_2_(NCS)” building units are arranged in the direction of the crystallographic b-axis with an antiparallel orientation of the tin–halogen bonds of neighbouring chains (Figure 7).

**Figure 8:**
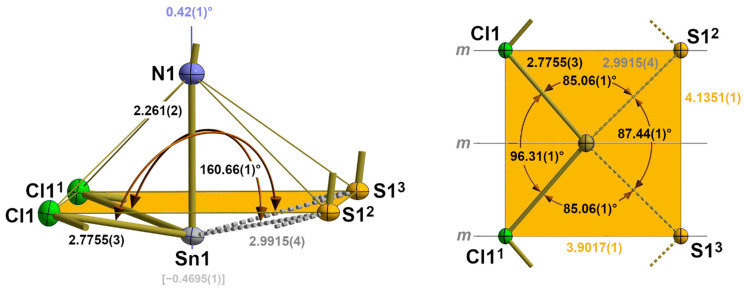
Side view (left) and top view (right) of the second coordination sphere of the tin atom in **2** with representative bond lengths [Å] and angles; distance (square brackets) of the tin atom below the basal plane (orange) of the quadrilateral pyramid; angle (blue value) between the tin–nitrogen bond and the normal vector of the basal plane. Symmetry transformations used to generate equivalent atoms: (^1^) x, 1 + y, z; (^2^) 1 − x, ½ + y, 1 − z; (^3^) 1 − x, −½ + y, 1 − z.

Including the interchain contacts based on the shortest tin–sulphur distances, the second coordination sphere of the tin atoms corresponds to a rectangular pyramid with the nitrogen atom of the isothiocyanate ligand at the apex and the tin atom below the exact planar basal plane formed by two halogen and two sulphur atoms (Figure 8).

While the tin–halogen distances of the first coordination sphere (2.1.2.) increase with increasing size of the halogen atom, the tin–sulphur distances of this second coordination sphere are of almost identical lengths [mean value: 2.996(12) Å] and show no systematic dependency on the size of the halogen atoms (Table 4).

**Figure 9:**
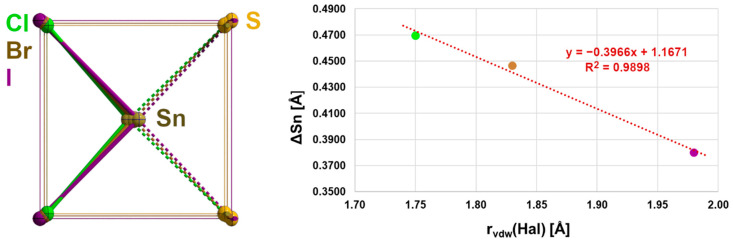
Overlay (**left**) of the second coordination spheres in **2**–**4** looking at the centres of the basal planes as a common focus; the bonds of first and second coordination spheres are drawn in the colours of the halogen atoms; graphical representation (**right**) of the ΔSn dependency on the van der Waals radius of the halogen atom with results of the linear regression analysis (red).

The height ΔSn (=distance of the tin atom from the basal plane) of the quadrilateral pyramid, however, decreases with increasing size of the halogen atoms from 0.46958(1) Å for Hal = Cl to 0.3799(6) Å for Hal = I (Table 4). Again, this effect is strongly correlated with the size of the halogen atom. In the basal plane, the tin···tin and sulphur···sulphur distances simultaneously increase with the increasing length of the b-axis in such a way that the rectangular plane becomes more and more square while the tin atoms move in a direction above the centre of the basal plane (Figure 9).

In order to classify the tin and sulphur distances in terms of bonds, they must first be compared with the values that describe a classical 2e-2c bond on the one hand and a situation in which there is probably no interaction between them on the other. Here, they are about 23% longer than the sum (2.44 Å) of the covalent radii of both elements but about 25% shorter than the sum (3.97 Å) of their van der Waals radii, which indicates a strong covalent character of these bonds whenever a 2c-2e bond is excluded (octet rule).

Regarding the symmetrical 3c-4e bond for the tin–fluorine interaction in the first coordination sphere of the tin atom in **1**, the covalent interaction between tin and sulphur in this α-SnCl(NCS) structure type is also suggested by the position of the sulphur atom in the *trans* position to a halogen atom, as it indicates the existence of a more or less asymmetrical 3e-4c Hal-Sn~S bond. The character of those three-centre bonds with different donor atoms X and Y is quantitatively difficult to record because of missing comparative data. As a benchmark for the Sn-S atom distance in a symmetrical 3c-4e S-Sn-S bond, however, a value of 2.838(4) [∠(S-Sn-S) = 145.2(2)°] is available [38]. Bond angles of the three-atom arrangement in **2**–**4** increase from 160.66(1)° for Hal = Cl to 165.60(2)° for Hal = I as the size of the halogen atom increases.

In terms of our terminology, the coordination mode of the tin atom changes in this α-SnCl(NCS) structure type from 3_0_ for the first coordination sphere to 3_2_-aa for the second one, whereby the letters “aa” designate the two asymmetric 3c-4e bonds.

**Figure 10:**
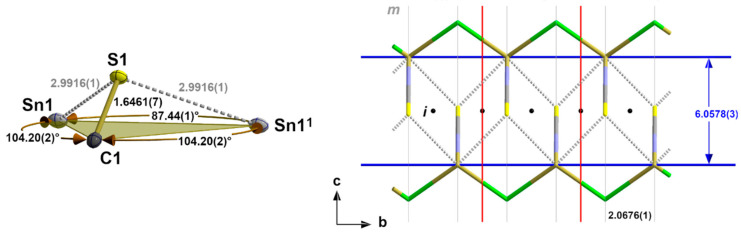
Ball-and-stick model (**left**) of the sulphur coordination in **2** with atom distances [Å] and angles; stick model (**right**) of the “zipper”-like arrangement of the primary building units as a result of the interchain tin–sulphur interactions with distances [Å] and positions of the symmetry elements (*m* = mirror plane, *i* = centre of symmetry); unit cell (red)); symmetry elements for the transformation of equivalent atoms: x, −1 + y, z.

Regarding the second Sn coordination sphere, the S atom adopts a *tpy* coordination with two tin atoms and the carbon atom in the basal plane (Figure 10). Although the halogen atoms are not involved in this sulphur coordination, some structural parameters show a strong halogen size dependency (Table 5). This particularly implies the bond angle between the two Sn atoms that increases by 4.99° from 87.44(1)° for Hal = Cl to 92.43(1)° for Hal = I. In summary, these interchain contacts result in a “zipper”-like arrangement (Figure 10, right) of the building units in **2**–**4**.

#### 3.2.3. β-SnCl(NCS), **5**

**Figure 11:**
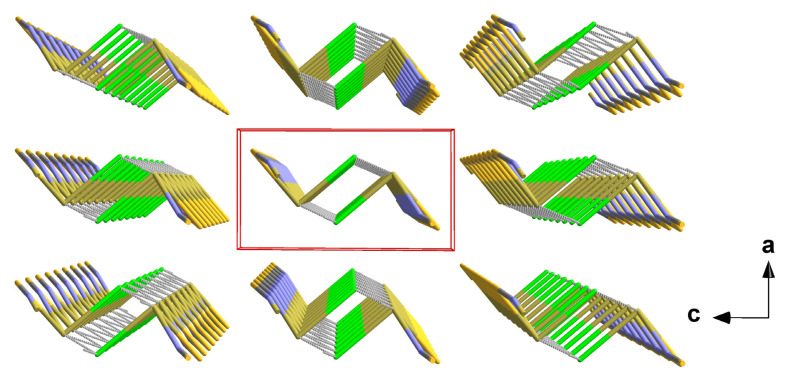
Perspective view of the crystal packing of **5**; stick model with unit cell presented in red looking down the b-axis. The interchain tin–sulphur contacts between neighbouring chains are indicated by dashed sticks in grey.

In **5**, the one-dimensional chains formed by the trigonal–pyramidal “Sn(μ_1_-Cl)(μ_2-_NCS)_2_” building units of the first coordination sphere (2.1.3.) are arranged in the direction of the crystallographic b-axis with the NCS dumbbells pointing in the same direction (Figure 11). Two different tin–chlorine contacts [3.1692(4)/3.2263(4) Å] that are significantly longer than the intrachain tin–chlorine distances [2.5501(4) Å] are exclusively responsible for the interchain interaction.

**Figure 12:**
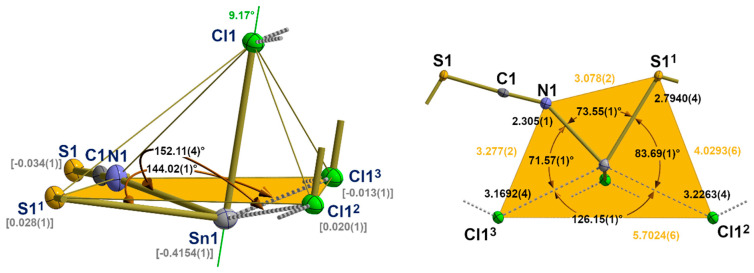
Ball-and-stick model of the second coordination sphere of the tin atom in **5** with representative atom distances [Å] and angles; distance (square brackets) of the tin atom below the least-squares plane of the basal atoms (orange) of the quadrilateral pyramid and the angle (green value) between the tin chlorine bond and the normal vector of the basal least-square plane. Symmetry transformations used to generate equivalent atoms: (^1^) x, −1 + y, z; (^2^) 1 − x, −y, 1 − z; (^3^) 1 − x, 1 − y, 1 − z.

With these two additional chlorine contacts, the tin atom achieves a fivefold, strongly distorted, quadrilateral coordination with a trapezoidal base (Figure 12). Distortions of this second coordination sphere mainly result from the non-planarity of the basal plane expressed by deflections of the least-plane constituting atoms of ±0.03 Å, different side lengths of the trapeze [3.078(2)–5.7024(6) Å], and the position of the apical chlorine atom whose bond to the tin atoms forms an angle of 9.17° with the normal vector of the least-square plane.

With respect to the structure–bonding relationship, the additional tin–chlorine contacts are in *trans* positions to a nitrogen and sulphur atom. The structural parameters of these three-centre X-Sn-Y arrangements are characterized by the following atom distances and angles: d(N-Sn) = 2.305(1) Å, d(Sn-Cl) = 3.2263(4) Å, ∠(N-Sn-S) = 152.11(4)°, and d(S-Sn) = 2.7940(4) Å, d(Sn-Cl) = 3.1692(4) Å, ∠(S-Sn-Cl) = 144.02(1)°, respectively. The angle between both additional basal chlorine atoms rises to 126.15(1)°.

Similarly to the changes in the coordination sphere of the tin atom in the α-SnCl(NCS) structure type, the change in the coordination mode of the tin atom in this β-SnCl(NCS) structure type takes place from |3_0_|^1^ of the first coordination sphere to |3_2_-aa|^2^ of the second one.

**Figure 13:**
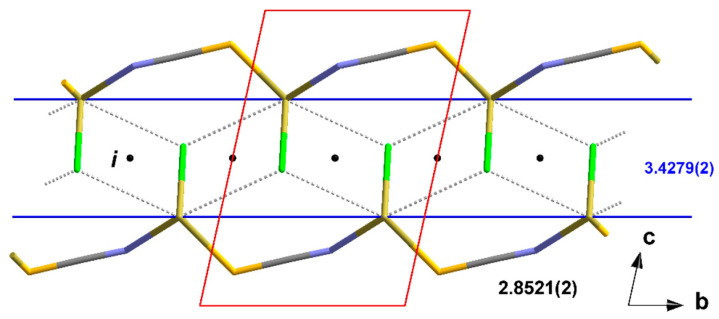
Stick model of the “zipper”-like arrangement of the atoms in **5** as a result of the interchain tin–chlorine interactions with characteristic distances [Å] between side parts and chain links; *i* = centres of symmetry; unit cell presented in red; symmetry elements for the transformation of equivalent atoms: (^1^) x, −1 + y, z.

The chlorine atoms adopt a *tpy* coordination, while the sulphur atoms adopt a twofold, bent coordination if all their contacts with the tin atom are taken into account as a result of the “zipper”-like arrangement (Figure 13) of the building units and the chlorine atoms act as linkers between both side parts (Sn atoms). The coordination spheres of the chlorine and sulphur atoms are presented together with their weak contacts in Figures S6 and S7, respectively.

### 3.3. Weak Interactions

Up to this point, the interatomic interactions within the first coordination sphere have been attributed to strong covalent 2c-2e and symmetric 3c-4e bonds, while the less strongly bonded atoms, which are added in the second coordination sphere, are interpreted as subordinate bond components in asymmetrical 3c-4e bonds, as they geometrically present themselves as extensions of symmetrical 3c-4e bonds and the tin atoms follow the octet rule when all bonds are taken into account.

However, it turns out that there are also much weaker contacts between “ladders” and “zippers”, which are ultimately responsible for their three-dimensional stacking in the crystal structures described here. These long-range interactions are, so to speak, the “glue” that packs “ladders” and “zippers” together.

As for many other tin(II) compounds, the statements made at the beginning regarding the hemi-directed coordination sphere and its rearward filling by additional long-range contacts also apply to the three structure types described here because such weak contacts on the backside of the covalent bonds are also found in all structures described here (Table 6), resulting in coordination numbers (CNs) seven and eight, respectively.

Regarding the structure–bonding relationship, such long-range contacts as a result of weak interactions are attributed in the literature to non-covalent interactions. In many other solid-state structures of p-block elements, especially those of higher periods, such weak, long-range interactions are observed with similar geometrical restrictions. They are referred to with group-specific terms like tetrel [39,40,41,42,43], pnictogen [42,43,44], chalcogen [42,43,45,46], and halogen bonds [43,47], or with element-specific terms like stibium bonds [48]. In this respect, the weak interactions considered here fall under the generic term tetrel or stannum bonds. As in some aspects, the stannum bonds observed here (interaction with p-orbitals exclusively) differ from those in tetravalent tin compounds (interactions with sp^3^ hybrid orbitals under rehybridization [41]), one is inclined to distinguish between stannous and stannic bonds.

Theoretically, such weak interactions are usually attributed to a region of low electron density, the so-called σ-hole, which is observed in covalently bonded atoms of the heavier p-block elements in the back extension of the covalent bond [49].

Although these long-range distances d(Sn···Y) can be quantified, for example, by use of the so-called normalized contact values [41] *N_c_* = d(Sn···Y)/B_vdW_, the ratio between the interatomic distance and B_vdW_ of Sn and Y, it is advised to compare them with distances d(Sn-Y) in case Y is covalently bound to the tin atom.

**Figure 14:**
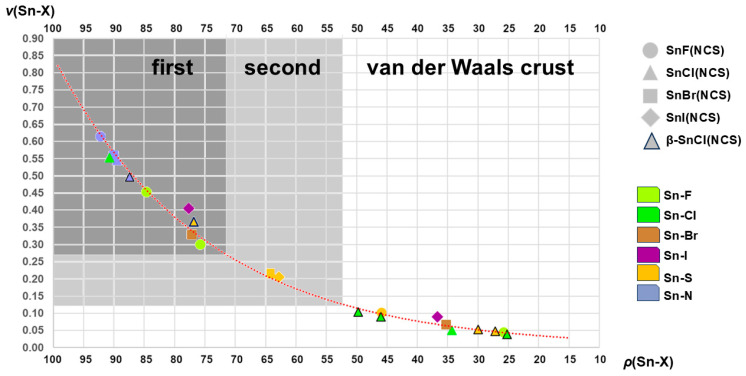
Graphical representation of the relationships between the interpenetration indices ρ and bond valences v for the complete list of interatomic distances Sn-X found in **1**–**5**; areas of atom distances that were found in this work in the first, second, and van der Waals crust coordination spheres are marked as dark grey, grey, and white fields; the dotted curved line in red shows the course of the exponential trend line expressed by the formula *v* = 0.0156 e ^0.0399*ρ*^ and the reliability index R^2^ = 0.9919.

This can be achieved by the use of the semi-quantitative calculation of bond valences, *v* [50], that are usually used in the calculation of bond valence sums, BVS, in order to determine the oxidation state of the corresponding atom on the one hand and interpenetration indices [51], *p*, on the other. The corresponding calculations of all contacts described in this study are summarized in Table S2 and visualized in Figure 14.

Although the parameters for *p* and *v* are based on different experimental data, they express the relationships between strong and weak interactions observed in the SnHal(NCS) structures quite similarly (Figure 14), an effect that is also reflected in the exponential course of the trend line. Both variables are obviously appropriate to describe the relationship between atom distance and bond strength.

This graph also shows that there is an almost continuous transition from strong to weak interactions between the tin atoms and their different ligand atoms without the different strengths of the interactions being sharply defined. Despite this uncertainty, the atomic distances used to describe the first and second coordination spheres are obviously correctly recorded, and the long-range interatomic distances of the van der Waals crust are delimited from them.

In addition, the representation of bond valences/interpenetration indices makes it clear that atom distances alone do not have to be decisive for the detection of the atoms of the first coordination sphere but that this must be converted into one of the two quantities in order to be able to make a valid statement. In this respect, this supports the assumptions regarding the first coordination sphere of **4** with d(Sn···S) < d(Sn-I), which was derived (2.1.2.) solely based on the fact that **4** is isostructural with **2** and **3**.

In another context, we found [28,52] that the atom distances d(Sn~X) and d(Sn~Y) in an X~Sn~Y three-centre arrangement, described by the asymmetry quotient Q = d(Sn-Y)/d(Sn-X), strongly correlate in the region of quasi-symmetrical 3c-4e bonds [Q < 1.05]. Although this correlation is less pronounced with increasing difference between the two atom distances [Q > 1.05], it seems reasonable to establish the threshold (TH) between the valence shell (the first and second coordination spheres) and the van der Waals crust (van der Waals crust sphere = region of tetrel bond) by use of two times the difference Δ_sym_ between the atom distances d(Sn-X) = d(Sn-Y), where Q = 1, and B_cov_ is calculated according to

Δ_sym_ = d(Sn-X)_sym_ − B_cov_, (1)

TH = B_cov_ + 2Δ_sym_ (2)

**Figure 15:**
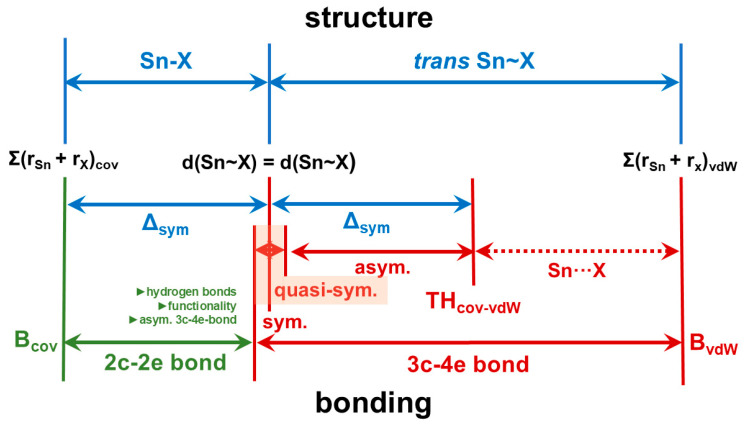
Structure–bonding relationships for the classification of strong/weak, covalent/non-covalent interactions in tin(II) compounds in terms of classical 2c-2e and various forms of 3c-4e bonds (sym = symmetrical [Q = 1], quasi-sym. = quasi-symmetrical [Q < 1.05], asym. = asymmetrical [1.05 < Q < 1.5]); factors that can influence the length of the 2c-2e bond are shown in dark green.

In the case of the μ_3_-F atom of **1**, for which this study provides a reliable value for d(Sn-X) = d(Sn-Y), Δ_sym_ amounts to 2.376–1.96 = 0.416 Å and TH to 1.96 + 2 × 0.416 = 2.792 Å. With respect to the interpenetration index, bond valence, and asymmetry quotient, this corresponds to a value of 65.05%, 0.13, and 1.42, respectively. For other donor atoms X, TH will deviate more or less from the value for μ_3_-F, but with respect to *p* and *v*, it will be in the same order of magnitude, resulting in the structure–bonding relationship as sketched out in Figure 15.

The greater the difference between the bond length d(Sn~Y) and the reference value (benchmark) of the 3c-4e bond, the smaller the overlap of the electron shells of tin and Y. If the difference is small and the overlap is, therefore, still large, the bond has the predominantly covalent character of an asymmetric 3c-4e bond, which decreases as the difference increases. If the difference is large and the overlap is, therefore, small, the input of electrons from Y into the 3c-4e bond is reduced, with the effect that the bond becomes electron-deficient and predominantly has the non-covalent character of an electrostatic tetrel bond.

From a geometrical point of view, long-range interactions always occur with lone-pair-processing atoms Y that are in the *trans* position to an atom X of the first coordination sphere. In this context, the term *trans* does not mean linear but bent, as angles (X-Sn···Y) are around 145 ± 15°, as Table 7 exemplarily shows in the case of the compounds discussed here. On the other hand, angles of similar magnitude are also found in the case that both atoms X and Y are exactly equidistant from the tin atom, representing the bonding situation described for a symmetrical 3c-4e bond. If quasi-symmetrical 3c-4e bonds are also considered, in which the atom distances Sn-X and Sn-Y are not identical but very similar [Q < 1.05] and in which similar angles occur, it becomes obvious that the long-range interactions discussed here are based on similar but more asymmetrical 3c-4e bonding situations, meaning that the octet rule is not violated.

The analogy between the 2e-3c bond [9] in the exactly symmetrical, linear hydrogen bond of the difluoride ion, [FHF]^-^, and the symmetrical but bent 3c-4e bond as found in **1** on the one hand and a classical, asymmetrical, non-linear hydrogen bond X-H···Y with a quasi-symmetrical or asymmetrical, bent three-centre bond X-Sn···Y, on the other hand, is obvious. It should, therefore, come as no surprise if bifurcations are possible with the latter, similar to bifurcations of canonical hydrogen bonds [53,54]. In the following, we will show that the weak, long-range contacts that are observed between “ladders” and “zippers” in all structures of the mixed halide isothiocyanate tin(II) compounds and which apparently cannot be reconciled with the octet rule because they exceed the coordination number 6 can be understood as extensions of asymmetric, bifurcated 3c-4e bonds.

#### 3.3.1. SnF(NCS)

**Figure 16:**
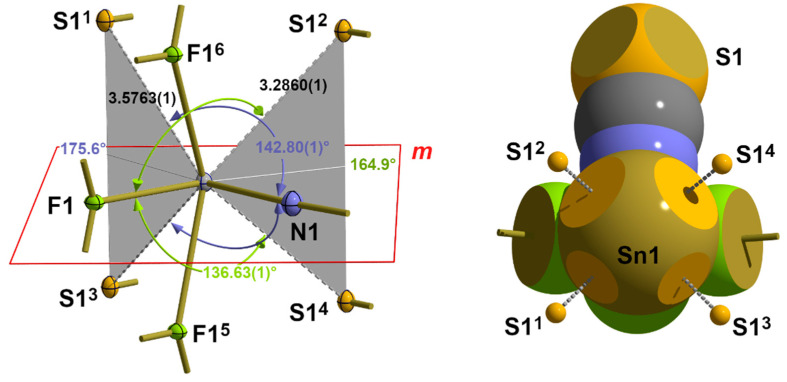
Ball-and-stick model (**left**) and space-filling model (**right**) with the position of the crystallographic mirror plane *m* and interatomic distances [Å] and angles (*trans* to F1 in green, *trans* to N1 in blue) describing the long-range Sn···S contacts in **1**; bifurcated tetrel bonds are indicated by grey planes and ABs; symmetry transformations used to generate equivalent atoms: (^1^) 1/2 − x, 1 − y, −1/2 + z; (^2^) 1 − x, 1/2 + y, 1 − z; (^3^) 1/2 − x, −y, −1/2 + z; (^4^) 1 − x, −1/2 + x, −1 − z; (^5^) −x, −1/2 + y, 1 − z; (^6^) −x, 1/2 + y, 1 − z.

In this structure type with the 3_1_-s, seesaw “SnF_4_” coordination mode (2.1.1.) at the tin atom, the space occupied by the four fluorine ligands is relatively small, leaving the electron shell of the tin atom on the side opposite the ligands largely unprotected for further interactions. In fact, in this area, four S atoms can be found belonging to the thiocyanate groups of neighbouring “ladders” at distances of 2 × 3.2860(1) and 2 × 3.5763(1) Å [Table 6]. Their *trans* position to the equatorial fluorine and nitrogen atoms of the |3_1_-s|^1^ coordination with the typical angles [142.80(1)°/136.63(1)°, Table 7] of an X-Sn···S arrangement is shown in Figure 16.

With four atoms of the first coordination sphere and four additional sulphur atoms from these van der Waals contacts, the tin atom achieves a coordination number of 8 and exceeds the magic number of six ligands, which the tin atom is able to bind via three 3c-4e bonds due to its three p-orbitals. It is noticeable, however, that one pair of sulphur atoms adopts a *trans* position to the fluorine atom, while another pair adopts a *trans* position to the nitrogen atom and that the two angle bisectors, ABs, between each pair of atoms are in the equatorial plane of the first coordination sphere. At the same time, the angles between the ABs and their *trans*-positioned atoms are 164.9° in the case of the fluorine atom and 175.6° in the case of the nitrogen atom. Using the gravity centres between each pair of symmetry-related sulphur atoms as additional virtual coordination points, in summary, a coordination number of 6 is provided for a distorted octahedron, which corresponds to a coordination polyhedron based on three 3c-4e bonds. All of this together points to a strongly asymmetric, bifurcated 3c-4e bonding situation.

According to our terminology, the complete coordination of the tin atom of **1** can be denoted as |3_3_-sa_b_a_b_|^3^, with the index “b” as an abbreviation for bifurcated and the superscript “3” indicating the van der Waals crust coordination sphere. In this way, the actual coordination number (8) can be easily extracted from this term by adding the number of ligand atoms represented by s (two atoms) and 2 x a_b_ (three atoms).

At the site of the sulphur atom, which is actually only bound to the carbon atom via a covalent 2c-2e single bond, the long-range interactions with the neighbouring tin atoms result in a rectangular–pyramidal coordination with the carbon atom at the apex and four tin atoms in the basal plane (Figures S7 and S8), which is exactly planar because of symmetry.

#### 3.3.2. α-SnCl(NCS), **2**, SnBr(NCS), **3**, SnI(NCS), **4**

**Figure 17:**
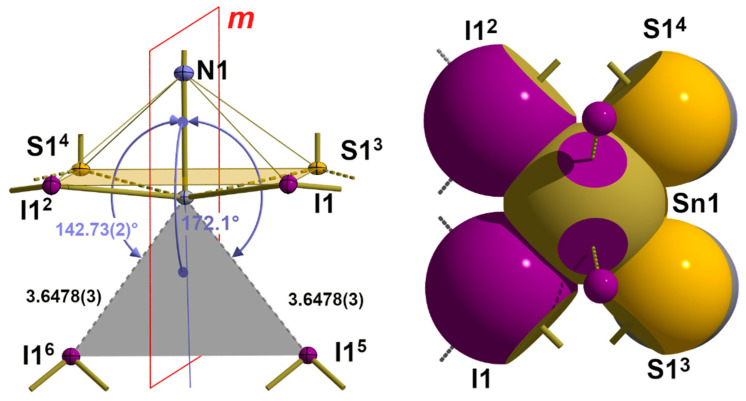
Ball-and-stick model (**left**) with the crystallographic mirror plane *m* and interatomic distances [Å] and angles of the long-range tin···iodine contacts as an example of these interactions in the α-SnCl(NCS) structure type; bifurcated tetrel bond indicated as a grey plane; angle bisector AB presented in blue; space-filling model (**right**) visualizing these contacts on the back of the tin–nitrogen bond; symmetry trans-formations used to generate equivalent atoms: (^2^) x, 1 + y, z; (^3^) 1−x, −1/2 + y, 1−z; (^4^) 1−x, 1/2 + y, 1−z; (^5^) 1/2 + x, 1/2−y, 1/2−z; (^6^) 1/2 + x, 3/2 + y, 1/2−z.

In this structure type with its |3_2_-aa|^2^ coordination mode (2.2.2.) at the tin atom, the space on the back of the bonds is much more restricted than in **1**. In fact, there are only two halogen atoms in the area in question that are involved in intermolecular interactions due to their distance and position relative to the tin atom. Both additional halogen atoms are mapped onto each other via a crystallographic mirror plane, whereby the tin···halogen distances increase from 3.4461(4) to 3.6478(3) Å due to the different sizes of the halogen atoms (Table 6). With bond angles of 141.21(1)–142.73(2)° (Table 7), these halogen atoms are *trans* to the apical nitrogen atoms of the isothiocyanate ligands in the second coordination sphere (Figure 17). Substitution of the two additional halogen atoms by their centre of gravities provides a virtual coordination point that reduces the coordination number from 7 to 6 in an always octahedral environment. In this bifurcated tetrel bond, both branches are of equal length, as in **1**, while the angle between the AB and the tin–nitrogen bond rises to 172.13(2)°. In summary, the van der Waals crust coordination sphere at the tin atoms can be denoted as |3_3_-aaa_b_|^3^.

As mentioned above, the tetrel bonds described here have many features in common with bifurcated hydrogen bonds. Accordingly, they are best described not only in terms of the subordinate parts of the 3c-4e bonds but—as for hydrogen bonds—with the complete set of atom distances and angles in the 3c-4e N-Sn···Hal arrangement. As an example, for this kind of data processing in the case of tetrel bonds, the corresponding data are summarized in Table 8 for the three isostructural compounds of the α-SnCl(NCS) structure type.

Combining the μ_2_-bridging interactions of the first coordination sphere with the additional two weak contacts of the van der Waals crust results in a square–pyramidal coordination of the halogen atoms. The structural changes in their coordination geometry as a function of the halogen size are listed in Table 9 and graphically visualized in Figure S9.

#### 3.3.3. ß-SnCl(NCS), **5**

**Figure 18:**
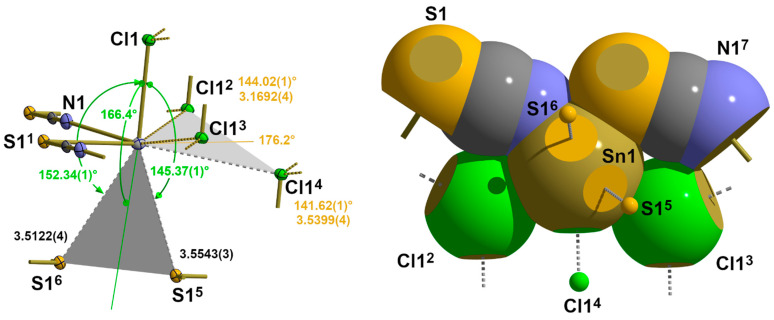
Ball-and-stick model (**left**) with atom distances [Å] and angles and space-filling model (**right**, viewing direction along the apical Sn-Cl bond) visualizing the geometrical details of the four long-range contacts in **5**; contacts *trans* to the nitrogen atom labelled in green, those *trans* to the sulphur atom in orange, and bifurcated tetrel bonds indicated as grey planes; symmetry operation used to generate symmetry related atoms: (^1^) x, y−1, z; (^2^) −x + 1, −y + 1, −z + 1; (^3^) −x + 1, −z, −z + 1; (^4^) x−1, y, z; (^5^) x−1, y−1, z; (^6^) −x + 1, −y + 1, −z + 2; (^7^) x, −1 + y, z.

According to the observed long interatomic distances (Table 6) in this second modification of SnCl(NCS), there are three contacts, two of sulphur atoms and one of a chlorine atom in adjacent “zippers”, on the backside of the |3_2_-aa|^2^ coordination sphere of the tin atom (Figure 18).

Both sulphur atoms are in a *trans* position to the *apical* chlorine atom, while the additional chlorine contact is found to be *trans* to the *basal* nitrogen atom. The geometrical details of these contacts are fully in line with the weak contacts of the previous structure types in terms of both distances and angles (Table 6 and Table 7).

As in the previous structures, these contacts can be interpreted as bifurcated tetrel bonds. Because of the lower symmetry of the tin site, both branches of these bifurcations are, however, of different lengths. For the bifurcation *trans* to the chlorine atom, this difference is small [0.0421 Å], but for the bifurcation *trans* to the sulphur atom, the difference is considerable [0.3647 Å], with one short branch (originally interpreted in 2.2.3. as an asymmetric 3c-4e bond) and one long one. In both cases, the angles between the ABs and the atoms in *trans* position are nearly linear [166.40(1)°/176.24(1)°].

Concerning the coordination behaviour of the sulphur and chlorine atoms, distorted tetrahedral coordination is observed if the tetrel bonds are taken into account in addition to their 2c-2e bonds. These coordination geometries are visualized in Figures S10 and S11, respectively.

**Figure 19:**
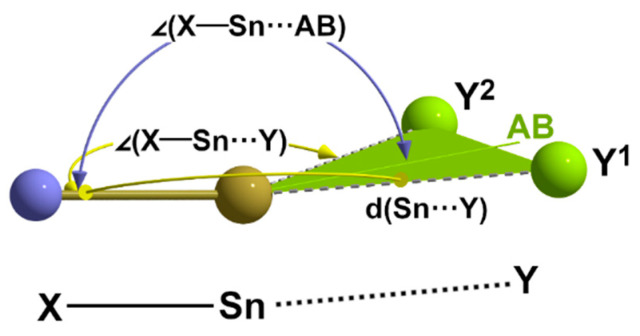
Schematic representation of the main structural parameters defining a bifurcated tetrel bond to two donor atoms Y^1^ and Y^2^; AB = angle bisector.

In summary, it can be stated that the weak interactions described here always occur in a *trans* position to an atom of the first coordination sphere and that the angles X-Sn-Y have values in the same order of magnitude as the angles in the case of symmetrical 3c-4e bonds. Whenever two weak contacts occur in a *trans* position to one and the same atom, the angle bisector between these atoms practically lies in line with the bond to which these atoms are *trans*. Together with the requirement of the octet rule, these geometrical restrictions schematically presented in Figure 19 suggest that these pairs of tetrel bonds are two branches of bifurcated, electron-deficient 3c-4e bonds.

## 4. Materials and Methods

### 4.1. Sample Preparation and Single Crystal Growth

For large-scale preparation, elemental analysis, crystallographic, and selected spectroscopic data of **1**–**4**, see Al Oraibi et al. [27]. Single crystals of **1**–**5** used in this study were grown in microscale experiments by adding a few drops of a sodium thiocyanate solution onto solid tin(II) dihalide, SnHal_2_, in a Petri dish, and crystal growth was observed by means of light microscopy. Both modifications of SnCl(NCS) crystallized side by side in the same experiment, with **2** predominating. Its crystals are needle-shaped, while those of **5** are rod-shaped.

### 4.2. X-Ray Diffraction and Structure Refinement

Single crystals suitable for X-ray measurements were selected under a microscope and mounted on a 50 μm MicroMesh MiTeGen Micromount^TM^ using a FROMBLIN Y perfluoropolyether (LVAC 16/6, Aldrich). Subsequently, they were centred on a Bruker Kappa APEX II CCD-based 4-circle X-ray diffractometer employed with graphite monochromated Mo Kα radiation (λ = 0.71073 Å) sourced from a fine-focus molybdenum-target X-ray tube operating at 50 kV and 30 mA. The crystal-to-detector distance was 40 mm, and the scan width was 0.5°. Cooling of the single crystals at 100(2) K was achieved using a Kryoflex low-temperature device. Details on the strategy for data collection and structure refinement have been published previously [55].

Further details of the crystal structure investigations may be obtained from the joint CCDC/FIZ Karlsruhe online deposition service (www.ccdc.cam.ac.uk/structures, accessed on 1 June 2025) by quoting the deposition numbers given in Table 1. Full lists of atomic coordinates, thermal displacement factors, bond lengths, and angles are given in the Supplementary Materials (Tables S1–S3 (**1**), Tables S4–S6 (**2**), Tables S7–S9 (**3**), Tables S10–S12 (**4**), and Tables S13–S15 (**5**)).

### 4.3. Figures

Figures were drafted by use of DIAMAND [56] and realized by means of POV-Ray [57]. In the ball-and-stick models, all atoms are drawn as thermal displacement ellipsoids of the 40% level.

Throughout this paper, the bond classification scheme developed here is graphically visualized by the use of thick sticks for pure or predominantly 2c–2e bonds, thin sticks for both bonding partners in symmetrical 3c–4e bonds, and thin, dashed sticks in grey for weakly bonded atoms in asymmetrical 3c–4e bonds. In all figures, bonds to atoms that are outside the structure section under consideration are indicated by shortened sticks with a thickness according to their classification.

Atoms are visualized in the space-filling models as single-coloured or truncated, two-coloured spheres according to their van der Waals radii and cut-offs based on the intersection of the two spheres with cut-off faces showing the colour of the interpenetrating atom. In order to better recognize the interactions between the tin atoms and the atoms that are attributed to the coordination sphere of the van der Waals crust, these are represented as small spheres with an arbitrary radius. Colours and van der Waals radii (Å) of the different atoms are as follows: F = light green/1.47; Cl = green/; Br = brown/; I = violet/; C = grey/1.70; N = blue/1.55; and Sn = brass/2.17. Bonds are indicated as shortened sticks of variable thickness and completeness according to the bonding classification scheme defined in this paper.

### 4.4. Calculations

B_cov_(Sn-X) = r_cov_(Sn) + r_cov_(X) and B_vdW_(Sn-X) = r_vdW_(Sn) + r_vdW_(X) were calculated using the r_cov_/r_vdW_ values Sn = 1.39/2.17, N = 0.71/1.55, F = 0.57/1.47, Cl = 1.02/1.75, Br = 1.20/1.83, I = 1.39/1.98, and S = 1.05/1.80 of Codero et al. [35] and Mantina et al. [31]. These values were also used for the calculation of the interpenetration indices *p*(Sn-X) = (d(Sn-X)_obs_ -B_cov_)/(B_vdW_-B_cov_) × 100. Bond valences *v*(Sn-X) *=* exp((r_o_–d(Sn-X))/b) were calculated with the conventional b value of 0.37 Å and the r_o_ values estimated by Brese & O’Keeffe [58] for F (1.925 Å) and by Hu et al. [59] for Cl (2.330 Å), Br (2.500 Å), I (2.572 Å), N (2.046 Å), and S (2.434 Å).

Since angle bisectors/gravity centres are not directly accessible experimentally, they were calculated without standard deviations using DIAMOND [56].

## 5. Conclusions

During a systematic study on mixed halide isothiocyanate tin(II) compounds, SnHal(NCS), five different single crystals belonging to three different structure types have been grown and structurally characterized. The three different structure types reveal a great number of unique structural details with respect to the coordination of the divalent tin atom resulting from strong to weak tin–ligand interactions. In the following, only some aspects of overriding importance are summarized.

The strong interactions that define, in accordance with the octet rule, the atoms of the first coordination sphere include, in particular, classical 2c-2e bonds Sn-X, but also symmetrical 3c-4e bonds X-Sn-X, such as those found in SnF(NCS), whose geometrical details represent benchmarks for this kind of bond in the case of μ_3_-coordinated fluorine atoms. Using the example of this bond length, it is emphasised how this value can help to define the threshold TH_cov-vdW_ between the so-called valence shell and van der Waals crust for each kind of atom X bound to Sn. Moreover, the three isostructural compounds of the α-SnCl(NCS) structure type reveal the strong correlation of specific structure parameters like atom distances and angles with the size of the halogen atom.

Medium–strong interactions are always observed in a *trans* position to strong bonds of the first coordination sphere and define the additional atoms of the second coordination sphere. Taking bond angles [∠(X-Sn~Y)~145 ± 15°] and the octet rule into account, these contacts can be interpreted as quasi-symmetrical [Q < 1.05] or asymmetrical 3c-4e bonds [1.05 < Q < 1.5].

In all structure types examined in the present study, additional long-range contacts always occur pairwise in a *trans* position to one and the same strong bond of the first coordination sphere. Considering the octet rule, atom distances and bond angles in the three-centre arrangements [∠(X-Sn~Y)~145 ± 15°, ∠(X-Sn~AB) = 175 ± 5°], which are often counted among tetrel bonds, are regarded as bifurcated, electron-deficient extensions of asymmetrical 3c-4e-bonds.

## Figures and Tables

**Table 1:** Crystal data, data collection, structure refinement, and CSD numbers of **1**–**5**. Data of isostructural compounds are marked with a coloured background.

	SnF(NCS)	α-SnCl(NCS)	SnBr(NCS)	SnI(NCS)	β-SnCl(NCS)
	1	2	3	4	5
Temperature	100(2)				
Wavelength [Å]	0.71073				
2θ_max_	60°				
Empirical formula	C F N S Sn	C Cl N S Sn	C Br N S Sn	C I N S Sn	C Cl N S Sn
Formula weight	195.77	212.22	256.68	303.67	212.22
Crystal system	orthorhombic	orthorhombic			triclinic
Space group	*Pnma*	*Pnma*			*P* $\underline{\bar{1}}$
Unit cell dimensions					
a [Å]	7.5937(3)	7.6567(2)	7.7230(3)	7.9583(3)	4.5920(2)
b [Å]	4.3031(2)	4.1351(1)	4.2273(2)	4.3450(1)	5.7024(2)
c [Å]	11.9462(5)	13.2907(4)	13.6146(5)	14.1490(5)	8.4084(4)
α	90°	90°	90°	90°	77.502(2)°
β	90°	90°	90°	90°	89.191(2)°
γ	90°	90°	90°	90°	87.101(2)°
V [Å^3^]	390.36(3)	420.80(2)	444.48(3)	489.26(3)	214.68(2)
Z, Z′	4, ½	4, ½			1, ½
d_calc_ [g/cm^3^]	3.331	3.350	3.836	4.123	3.283
μ(MoK_α_) [mm^−1^]	6.891	6.988	15.005	11.776	6.848
F(000)	352	384	456	528	192
Reflections collected	23,696	56,867	55,406	55,420	9562
Reflections unique, R_int_	637, 0.0465	694, 0.0544	722, 0.0374	798, 0.0527	1253, 0.0286
Data/restraints/parameters	637/0/32	694/0/32	722/0/32	798/0/31	1253/0/47
Goodness-of-fit on F^2^	1.123	1.129	1.175	1.264	1.103
R_1_, wR2 [I > 2σ(I)]	0.0116, 0.0237	0.0101, 0.0254	0.0119, 0.0316	0.0151, 0.0379	0.0115, 0.0232
R_1_, wR2 [all data]	0.0136, 0.0242	0.0120, 0.0261	0.0120, 0.0316	0.0164, 0.0382	0.0122, 0.0234
Extinction coefficient	0.0025(3)	n/a	n/a	n/a	0.0222(8)
±Δe [eÅ^−3^]	0.331/−0.401	0.291/−0.570	0.571/−0.6.16	1.002/−1.174	0.446/−0.438
CSD numbers	2449526	2449528	2449529	2449530	2449531

**Table 2:** Bond lengths and angles in the first 3_0_-coordination sphere of **2** (Hal = Cl), **3** (Hal = Br), and **4** (Hal = I); ΔSn = distance of the tin atom from the basal plane of the trigonal pyramid.

Hal	d(Sn-Hal) [Å]	d(Sn-N) [Å]	∠(Hal-Sn-Hal)	∠(Hal-Sn-N)	ΔSn [Å]	∠(Sn-Hal-Sn) **
Cl	2.7755(3)	2.261(2)	96.30(1)°	80.53(3)°	1.594(1)	96.30(1)°
Br	2.9122(2)	2.271(3)	93.07(4)°	81.66(4)°	1.652(1)	93.07(1)°
I *	3.0865(3)	2.261(4)	89.48(1)°	84.10(7)°	1.684(2)	89.48(1)°

* d(Sn-I) > d(Sn···S), ** equivalent to (Hal-Sn-Hal) because of symmetry.

**Table 3:** Bond lengths and angles of the isothiocyanate ligands in **1**–**5**.

	Hal	μ		d(N-C) [Å]	d(C-S) [Å]	∠(Sn-N-C)	∠(N-C-S)
**1**	F	1	N	1.156(3)	1.640(2)	171.9(2)°	178.1(2)°
**2**	Cl	1	N	1.158(2)	1.646(2)	170.9(2)°	177.7(2)°
**3**	Br	1	N	1.154(4)	1.649(3)	170.5(2)°	178.2(3)°
**4**	I	1	N	1.155(5)	1.652(4)	170.8(3)°	178.2(4)°
	mean	1	N	1.156(2)	1.649(3)	170.7(2)°	178.0(3)°
**5**	Cl	2	*κ* ^2^ *NS*	1.165(2)	1.649(2)	144.8(1)°	177.5(2)°

**Table 4:** Main interatomic distances [Å] and angles characterizing the second coordination spheres of the tin atoms in the α-SnCl(NCS) structure type.

Hal	d(Sn-Hal)	d(Sn-S)	d(Sn-N)	∠(Hal-Sn-S)	ΔSn	d(Hal···S)	b
Cl	2.7755(3)	2.9915(4)	2.261(2)	160.66(1)°	0.4695(1)	3.9017(1)	4.1351(1)
Br	2.9122(2)	2.9880(5)	2.271(3)	162.51(2)°	0.4466(1)	4.0154(2)	4.2273(2)
I	3.0865(3)	3.0093(7)	2.261(4)	165.60(2)°	0.3799(6)	4.3450(1)	4.3450(1)

**Table 5:** Interatomic distances [Å] and angles of the sulphur atoms in **2**–**4** of the first and second coordination spheres of the tin atoms; ΔS = distance [Å] of the sulphur atoms from the basal plane.

Hal	d(S···Sn)	d(S-C)	∠(Sn···S···Sn)	∠(C-S···Sn)	ΔS	d(Sn···Sn) = b
Cl	2.9916(1)	1.646(2)	87.44(1)°	104.20(2)°	1.069(1)	4.1351(1)
Br	2.9880(1)	1.649(3)	90.04(1)°	104.39(1)°	1.051(2)	4.2273(2)
I	3.0093(7)	1.652(4)	92.43(3)°	103.44(1)°	1.058(3)	4.3450(1)

**Table 6:** Interatomic distances [Å] characterizing the long-range contacts in the crystal structures of **1**–**5**; CN = coordination number.

	SnF(NCS)	α-SnCl(NCS)	SnBr(NCS)	SnI(NCS)	β-SnCl(NCS)
CN_cov_	4	5	5	5	5
d(Sn···Hal)	-	3.4461(4)	3.5038(3)	3.6478(3)	3.5399(4)
	-	3.4461(4)	3.5038(3)	3.6478(3)	-
d(Sn···S)	3.2860(1)	-	-	-	3.5122(4)
	3.2860(1)	-	-	-	3.5543(3)
	3.5763(1)	-	-	-	-
	3.5763(1)	-	-	-	-
CN(_cov + weak)_	8	7	7	7	8

**Table 7:** Interatomic angles in the case of the weak Sn-Y (Y = Hal, S) interactions found in the crystal structures of **1**–**5**; CN = coordination number.

	SnF(NCS)	α-SnCl(NCS)	SnBr(NCS)	SnI(NCS)	β-SnCl(NCS)
CN_cov_	4	5	5	5	5
∠(N-Sn···Hal)	-	141.92(1)°	142.12(1)°	142.72(2)°	141.62(4)°
	-	141.92(1)°	142.12(1)°	142.72(2)°	-
∠(Cl-Sn···S)		-	-	-	152.34(1)°
		-	-	-	145.37(1)°
∠(F-Sn···S)	136.63(1)°	-	-	-	-
	136.63(1)°	-	-	-	-
∠(N-Sn···S)	142.80(1)°	-	-	-	-
	142.80(1)°	-	-	-	-
CN_(cov + weak)_	8	7	7	7	8

**Table 8:** Interatomic distances [Å] and angles characterizing the bifurcated tetrel bonds in **2**–**4** as a function of the halogen atom; AB = angle bisector.

Hal	d(Hal···Sn)	∠(N-Sn···Hal)	∠(N-Sn···AB)	∠(Hal···Sn···Hal)	d(Hal···Hal)
Cl	3.4461(4)	141.92(1)°	169.7°	73.73(1)°	4.1351(1)
Br	3.5038(3)	142.12(1)°	171.7°	74.20(1)°	4.2273(2)
I	3.6478(3)	142.72(2)°	172.1°	73.10(1)°	4.3450(1)

**Table 9:** Atom distances [Å] and angles within the square–pyramidal coordinated halogen atoms according to all their contacts within the van der Waals crust coordination spheres.

Hal	d(Hal···Sn)	d(Hal-Sn)	∠(Sn···Hal···Sn)	∠(Sn-Hal···Sn)	ΔHal	d(Sn···Sn) = b
Cl	3.4460(4)	2.7756(3)	73.74(1)°	88.61(1)°	0.7130(4)	4.1351(1)
Br	3.5037(3)	2.9122(2)	74.21(1)°	89.18(1)°	0.7867(3)	4.2230(2)
I	3.6478(3)	3.0865(3)	73.10(1)°	90.11(1)°	0.9066(3)	4.3450(1)

## Data Availability

Can be excluded.

## Supplementary Materials

The following supporting information can be downloaded at: https://www.mdpi.com/article/10.3390/molecules30132700/s1. Figure S1. Unit cell volume of 1–4 as a function of van der Waals radii; Figure S2: Unit cell parameters of 1–4 as a function of van der Waals radii; Figure S3. Tin–halogen distances in 2–4 as a function of the covalent radii; Figure S4. Distance ΔSn of the tpy coordinated tin atom in 2–4 as a function of van der Waals radii; Figure S5. Bond angles Hal-Sn-Hal at the tpy coordinated tin atoms in 2–4 as a function of covalent radii; Figure S6. Relation of d(Sn-Hal) to d(Sn···S) in 2–4; Figure S7. Ball-and-stick model of the rectangular–pyramidal coordination of the S atoms in 1; Figure S8. Space-filling model of the rectangular–pyramidal coordination of the S atoms in 1; Figure S9. Ball-and-stick and space-filling model describing the rectangular–pyramidal coordination of the halogen atoms in 2–4; Figure S10. Ball-and-stick and space-filling model describing the distorted tetrahedral coordination of the chlorine atom in 5; Figure S11. Ball-and-stick and space-filling model describing the distorted tetrahedral coordination of the sulphur atoms in 5; Table S1. Atomic coordinates for 1; Table S2. Bond lengths and angles for 1; Table S3. Anisotropic displacement factors for 1; Table S4. Atomic coordinates for 2; Table S5. Bond lengths and angles for 2; Table S6. Anisotropic displacement factors for 2; Table S7. Atomic coordinates for 3; Table S8. Bond lengths and angles for 3; Table S9. Anisotropic displacement factors for 3; Table S10. Atomic coordinates for 4; Table S11. Bond lengths and angles for 4; Table S12. Anisotropic displacement factors for 4; Table S13. Atomic coordinates for 5; Table S14. Bond lengths and angles for 5; Table S15. Anisotropic displacement factors for 5; Table S16. Calculated interpenetration indices and bond valence.

## Abbreviations

The following abbreviations are used in this manuscript:

AB	angle bisector
B_cov_	covalent boundary
B_vdW_	van der Waals boundary
BVS	bond valence sum
*N_c_*	normalized contact value
*p*	interpenetration index
Q	asymmetry quotient
TH_cov-vdW_	threshold
*tpy*	trigonal–pyramidal
*v*	bond valence
