# Supplementary material for: Mixed Halide Isothiocyanate Tin(II) Compounds, SnHal(NCS): Signs of Tetrel Bonds as Bifurcated Extensions of Long-Range Asymmetric 3c-4e Bonds
Source: Molecules. 2025 Jun 23;30(13):2700. doi: 10.3390/molecules30132700 (PMC12250633; doi:10.3390/molecules30132700)

checkCIF/PLATON report

Structure factors have been supplied for datablock(s) HR2112_0ma_a

THIS REPORT IS FOR GUIDANCE ONLY. IF USED AS PART OF A REVIEW PROCEDURE FOR PUBLICATION, IT SHOULD NOT REPLACE THE EXPERTISE OF AN EXPERIENCED CRYSTALLOGRAPHIC REFEREE.

No syntax errors found.   CIF dictionary   Interpreting this report

Datablock: HR2112_0ma_a

Bond precision: N- C = 0.0060 A   Wavelength=0.71073

Cell:	a=7.9583(3)	b=4.3450(1)	c=14.1490(5)
	alpha=90	beta=90	gamma=90
Temperature:	100 K		

	Calculated	Reported
Volume	489.26(3)	489.26(3)
Space group	P n m a	P n m a
Hall group	-P 2ac 2n	-P 2ac 2n
Moiety formula	C I N S Sn	?
Sum formula	C I N S Sn	C I N S Sn
Mr	303.69	303.67
Dx,g cm-3	4.123	4.123
Z	4	4
Mu (mm-1)	11.775	11.776
F000	528.0	528.0
F000'	524.16	
h,k,lmax	11,6,19	11,6,19
Nref	798	798
Tmin,Tmax	0.542,0.610	0.561,0.782
Tmin'	0.064	

Correction method= # Reported T Limits: Tmin=0.561 Tmax=0.782
AbsCorr = MULTI-SCAN

Data completeness= 1.000   Theta(max)= 29.961

R(reflections)= 0.0151(766)   wR2(reflections)= 0.0382(798)

S = 1.264   Npar= 31

The following ALERTS were generated. Each ALERT has the format
test-name_ALERT_alert-type_alert-level.
Click on the hyperlinks for more details of the test.

Alert level G

```
PLAT004_ALERT_5_G Polymeric Structure Found with Maximum Dimension          2 Info
PLAT040_ALERT_1_G No H-atoms in this Carbon Containing Compound ..     Please Check
PLAT794_ALERT_5_G Tentative Bond Valency for Sn1       (II)      .       1.89 Info
PLAT967_ALERT_5_G Note: Two-Theta Cutoff Value in Embedded .res ..       60.0 Degree
PLAT969_ALERT_5_G The 'Henn et al.' R-Factor-gap value ..........      4.206 Note
              Predicted wR2: Based on SigI**2  0.91 or SHELX Weight  3.02
```

0 **ALERT level A** = Most likely a serious problem - resolve or explain
0 **ALERT level B** = A potentially serious problem, consider carefully
0 **ALERT level C** = Check. Ensure it is not caused by an omission or oversight
5 **ALERT level G** = General information/check it is not something unexpected

1 ALERT type 1 CIF construction/syntax error, inconsistent or missing data
0 ALERT type 2 Indicator that the structure model may be wrong or deficient
0 ALERT type 3 Indicator that the structure quality may be low
0 ALERT type 4 Improvement, methodology, query or suggestion
4 ALERT type 5 Informative message, check

It is advisable to attempt to resolve as many as possible of the alerts in all categories. Often the minor alerts point to easily fixed oversights, errors and omissions in your CIF or refinement strategy, so attention to these fine details can be worthwhile. In order to resolve some of the more serious problems it may be necessary to carry out additional measurements or structure refinements. However, the purpose of your study may justify the reported deviations and the more serious of these should normally be commented upon in the discussion or experimental section of a paper or in the "special_details" fields of the CIF. checkCIF was carefully designed to identify outliers and unusual parameters, but every test has its limitations and alerts that are not important in a particular case may appear. Conversely, the absence of alerts does not guarantee there are no aspects of the results needing attention. It is up to the individual to critically assess their own results and, if necessary, seek expert advice.

Publication of your CIF in IUCr journals

A basic structural check has been run on your CIF. These basic checks will be run on all CIFs submitted for publication in IUCr journals (*Acta Crystallographica*, *Journal of Applied Crystallography*, *Journal of Synchrotron Radiation*); however, if you intend to submit to *Acta Crystallographica Section C* or *E* or *IUCrData*, you should make sure that full publication checks are run on the final version of your CIF prior to submission.

Publication of your CIF in other journals

Please refer to the *Notes for Authors* of the relevant journal for any special instructions relating to CIF submission.

PLATON version of 02/02/2025; check.def file version of 02/02/2025

Datablock HR2112_0ma_a - ellipsoid plot

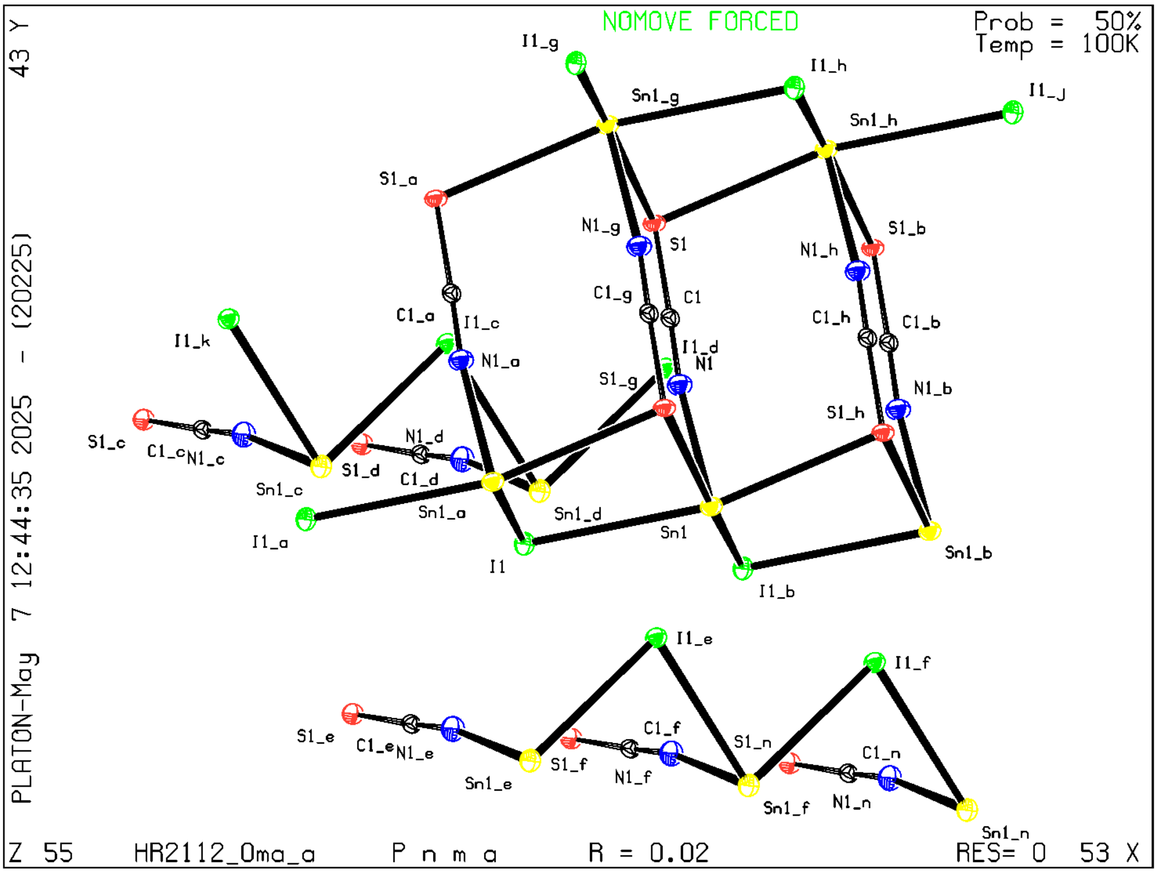

Supplement: Supplementary file 1 [file molecules-30-02700-s001.zip › molecules-3688367-supplementary/checkcif 4 SnI(NCS).pdf]
