# Supplementary material for: Mixed Halide Isothiocyanate Tin(II) Compounds, SnHal(NCS): Signs of Tetrel Bonds as Bifurcated Extensions of Long-Range Asymmetric 3c-4e Bonds
Source: Molecules. 2025 Jun 23;30(13):2700. doi: 10.3390/molecules30132700 (PMC12250633; doi:10.3390/molecules30132700)
Supplement: Supplementary file 1 [file molecules-30-02700-s001.zip › molecules-3688367-supplementary/Supplementary Material.docx]

Supplementary Material

*Mixed halide-isothiocyanate tin(II) compounds, SnHal(NCS): Tetrel bonds as bifurcated, electron-deficient extensions of symmetrical 3c-4e-bonds*

Hans Reuter

|  |
| --- |

Chemistry, Department of Biology/Chemsitry, Osnabrück University, Barbarastr. 7, D-49069 Osnabrück

Correspondence: [hreuter@uos.de](mailto:hreuter@uos.de)

## Figures


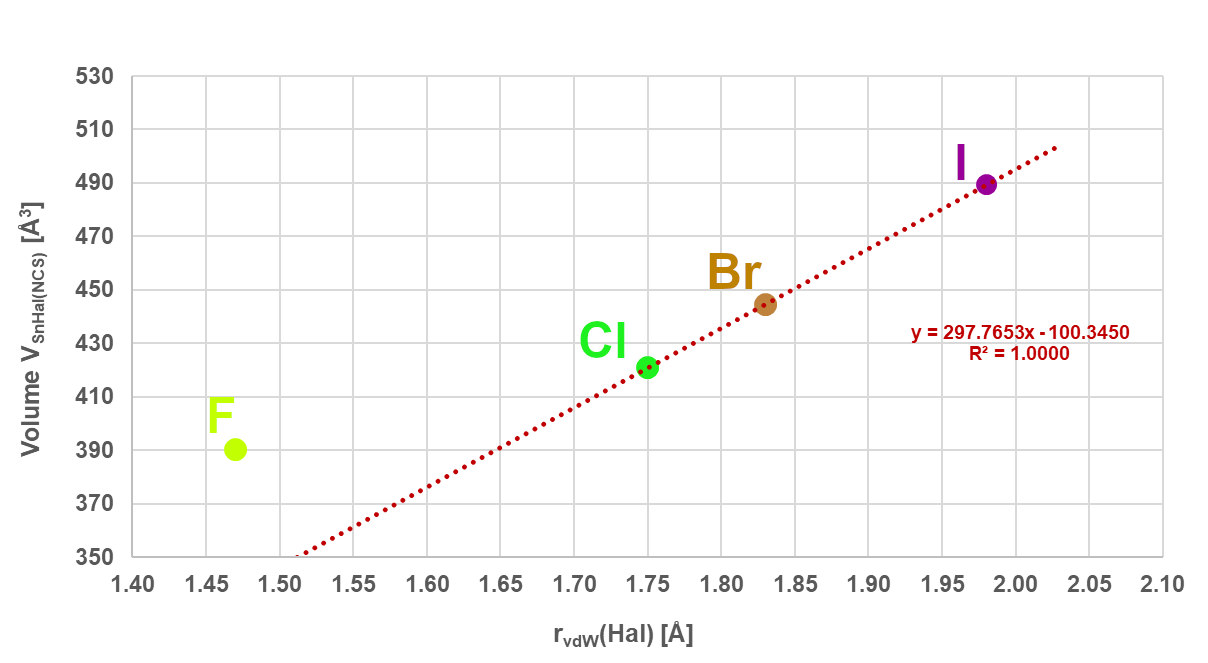


Figure S1: Unit cell volume in the series of the orthorhombic SnHal(NCS) structures, space group Pnma, as function of the van der Waals radii of the halide atoms, in red


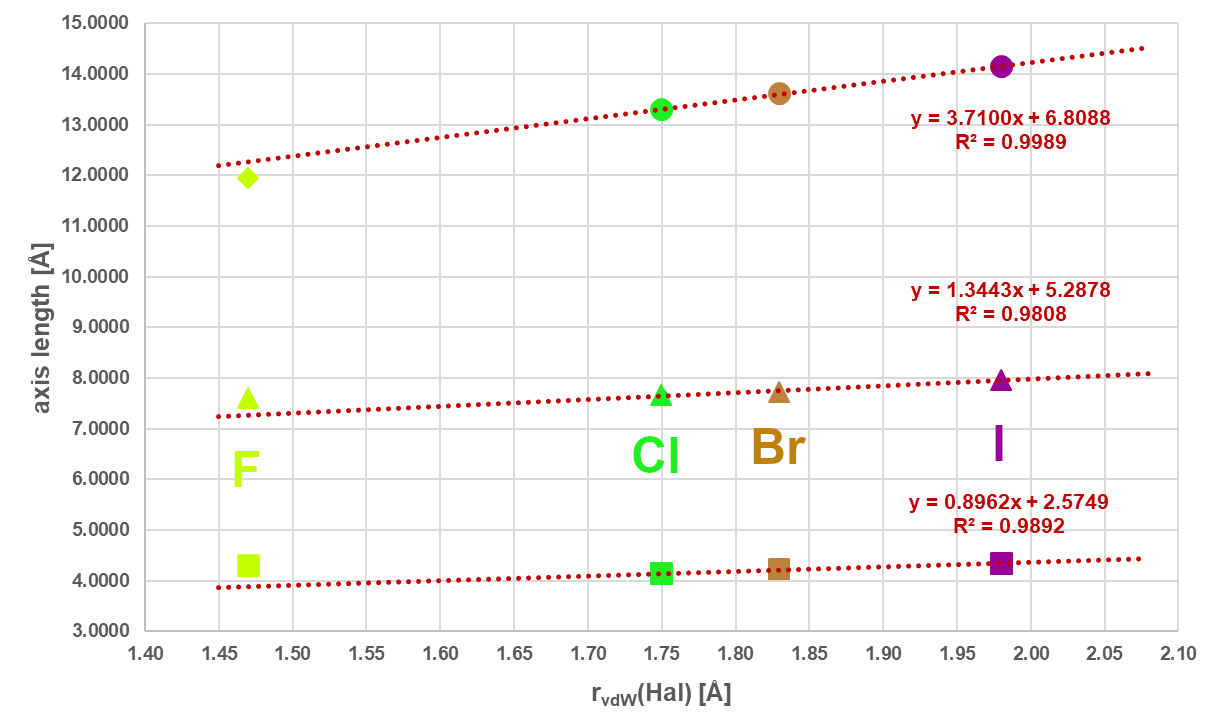


Figure S2: Parameters (a = triangles, b = squares, c = circles) of the unit cell of the orthorhombic SnHal(NCS), space group Pnma, as function of the van der Waals radii of the halide atoms; in red


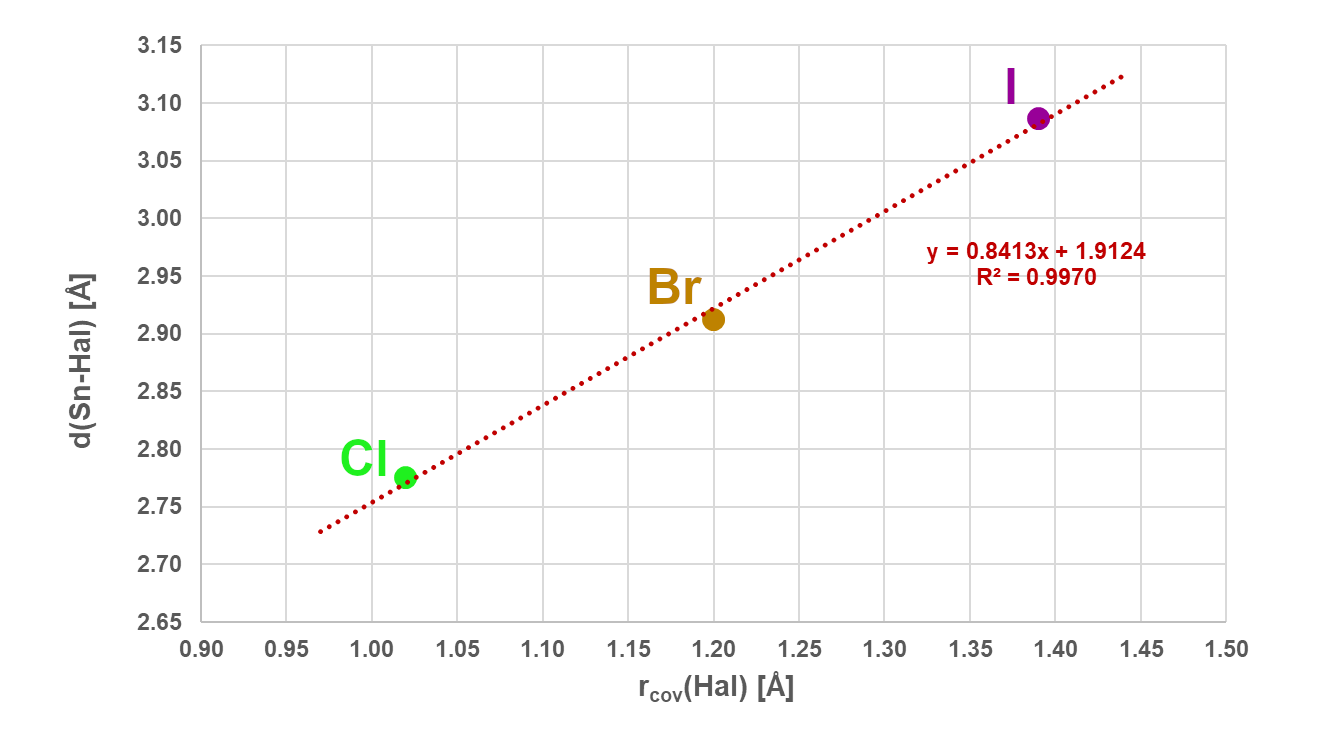


Figure S3: Tin-halide atom distances in 2 – 4 as function of the covalent radii of the halides Cl, Br, and I; formula and reliability index R^2^ of the linear regression analysis (dotted line) in red.


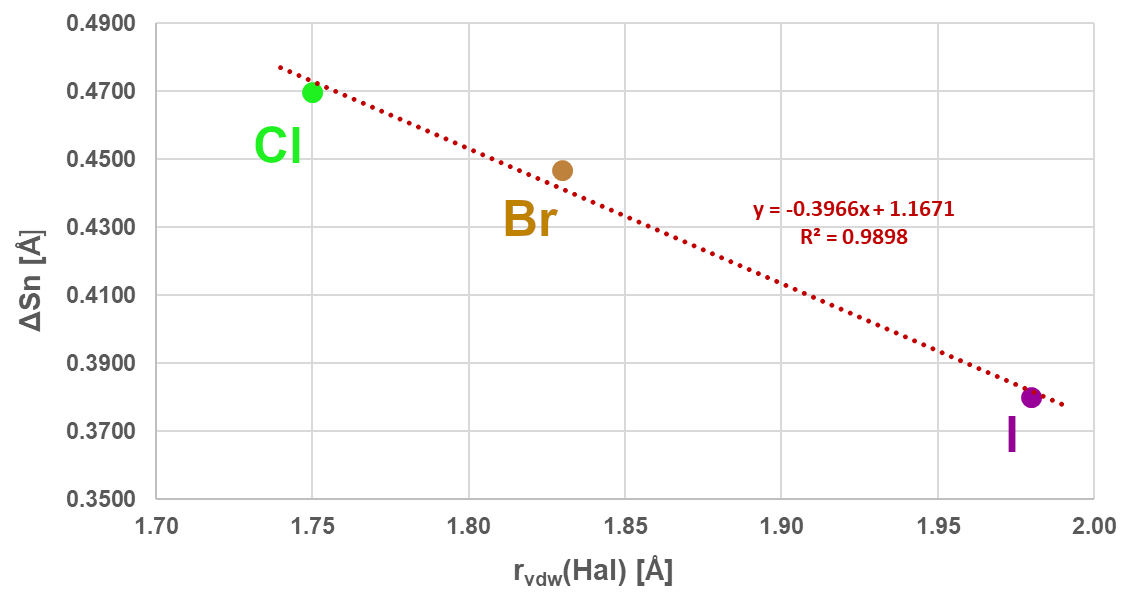


Figure S4: Distance ΔSn af the trigonal-pyramidal tin atoms in 2 - 4 from the basal plane as function of the van der Waals radii of the halides Cl, Br, I; formula and reliability index R^2^ of the linear regression analysis (dotted line) in red.


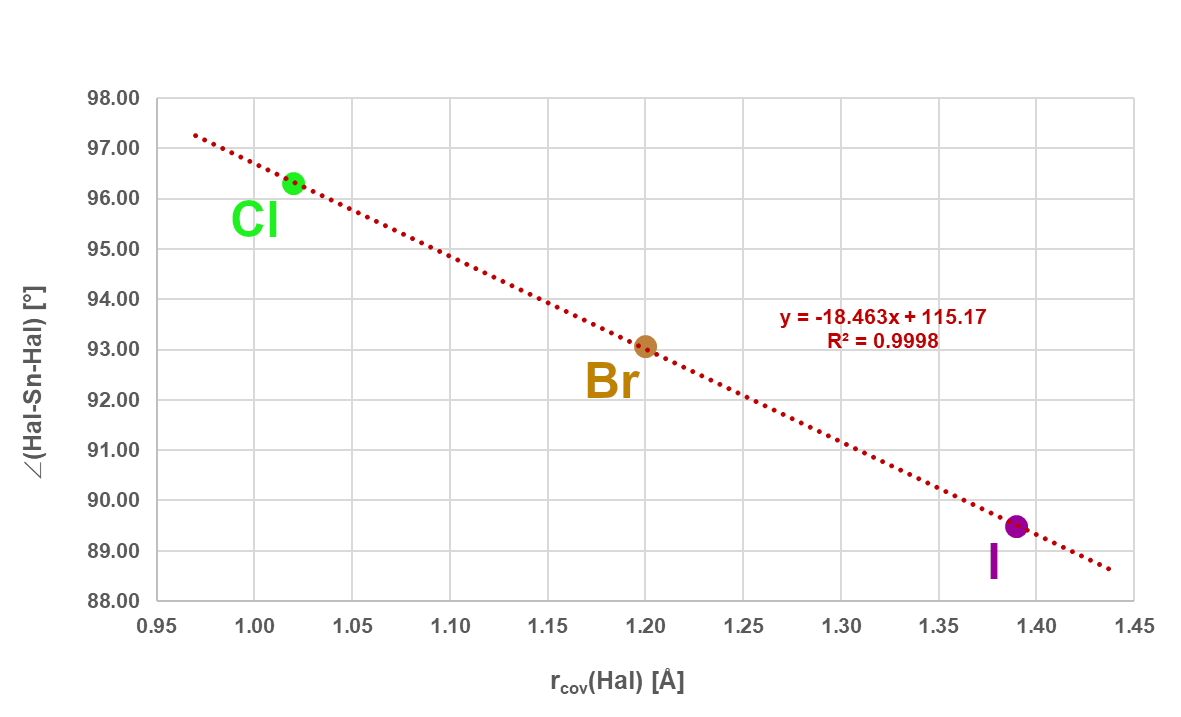


Figure S5: Bond angles Hal-Sn-Hal) at the trigonal-pyramidal coordinated tin atoms in **2** – **4** as function of the covalent radii of Cl, Br, and I; formula and reliability index R^2^ of the linear regression analysis (dotted line) in red.


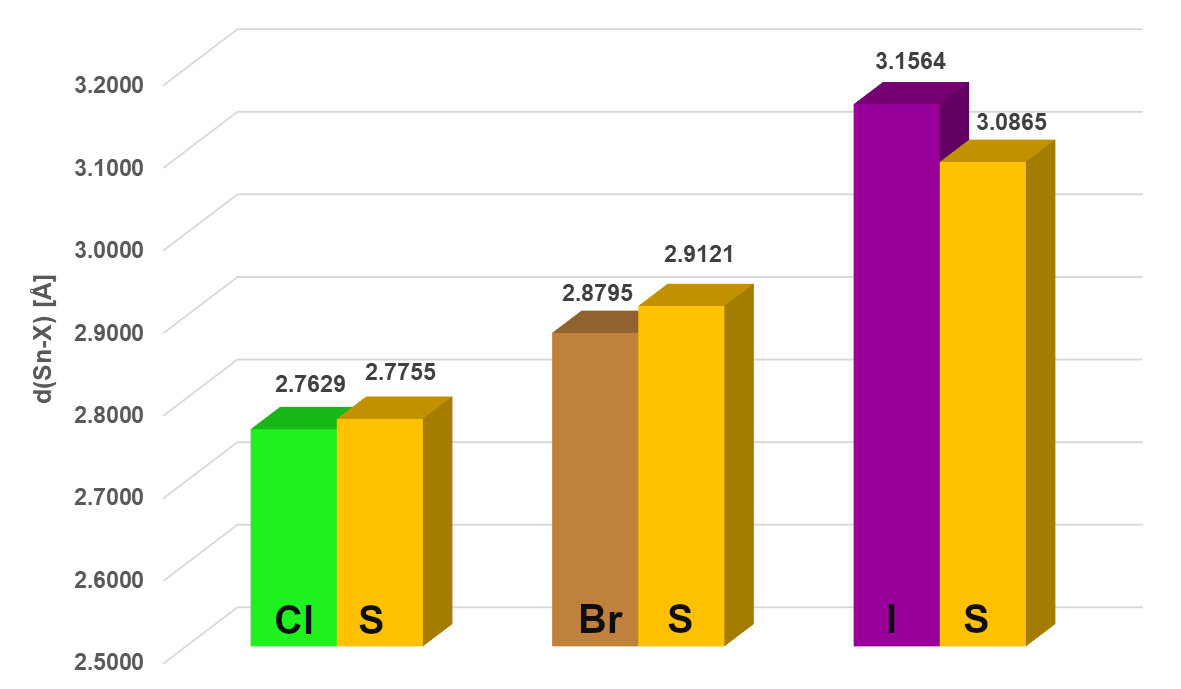


Figure S6: Relation of d(Sn-Hal) to d(Sn···S) in **2** - **4**.


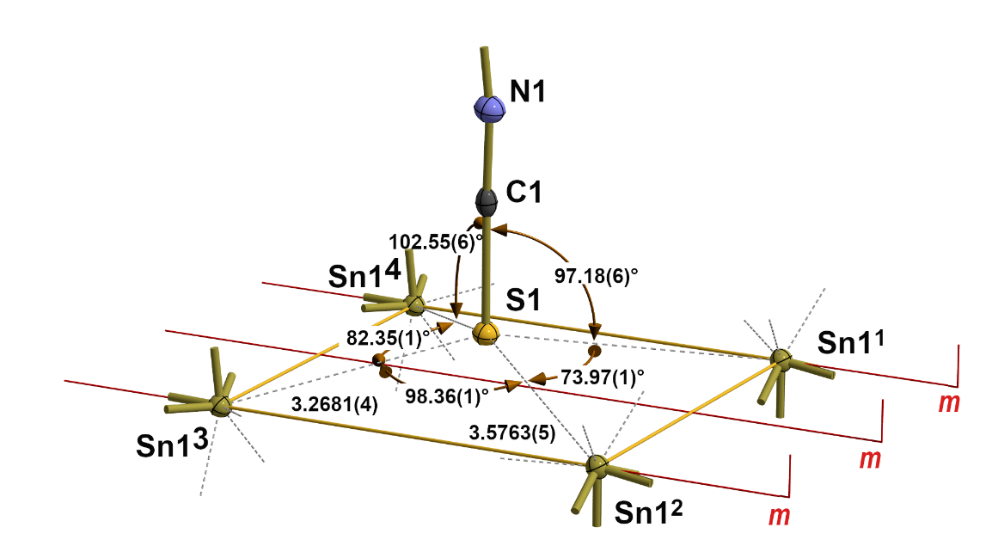


Figure S7: Ball-and-stick model with interatomic distances [Å] and angles describing the rectangular-pyramidal tin environment of the sulphur atom in **1**, crystallographic mirror planes m marked in red; symmetry operations used to generate equivalent atoms: ^1^) ½-x, 1-y, ½+z; ^2^) ½-x, -y, ½+z; ^3^) 1-x, -½+y, 1-z; ^4^) 1-x, ½+y, 1-z.


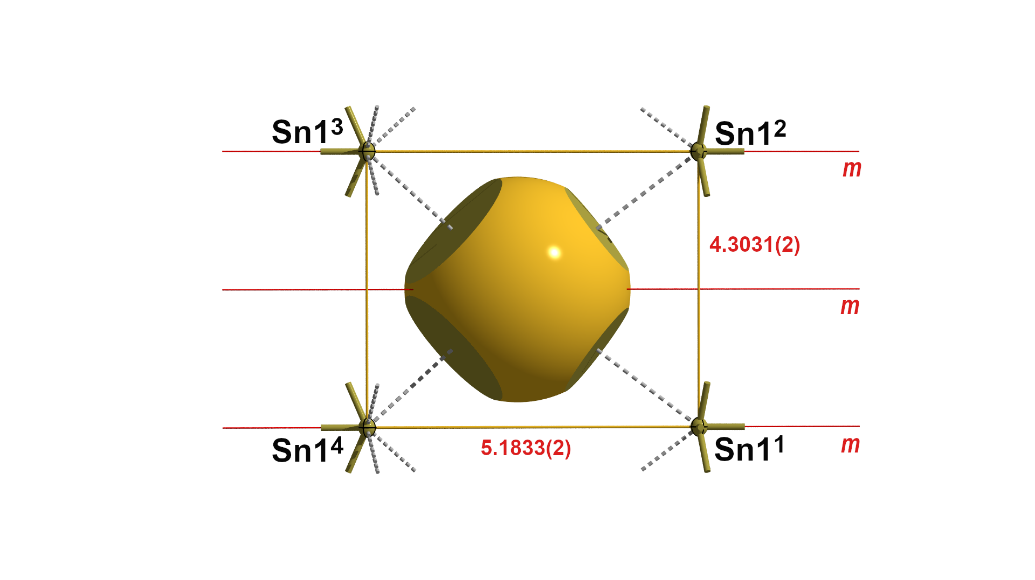


Figure S8: Space-filling model with crystallographic mirror planes m (red) and tin-tin distances [Å] describing the rectangular-pyramidal tin environment of the sulphur atom in **1**, crystallographic mirror planes m and tin-tin distances [Å] marked in red; symmetry operations used to generate equivalent atoms: ^1^) ½-x, 1-y, ½+z; ^2^) ½-x, -y, ½+z; ^3^) 1-x, ½+y, 1-z; ^4^) 1-x, ½+y, 1-z.

**
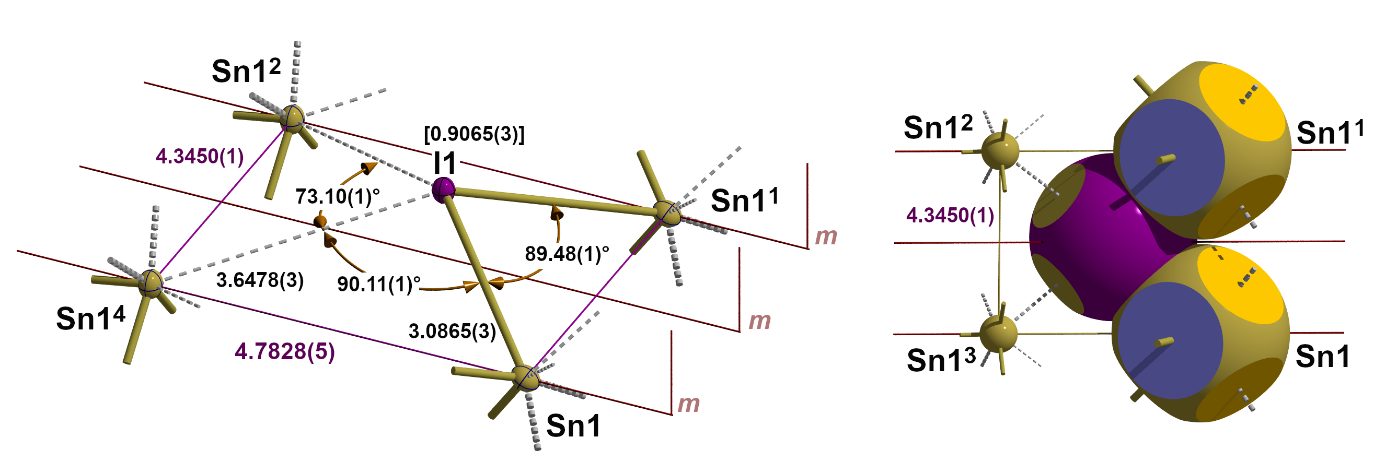
**

Figure S9: Ball-and-stick model (l**eft**) and space-filling model (**right**) with interatomic distances [Å] and angles describing the pseudo rectangular-pyramidal tin environment of the halide (here Hal = I) in **2** – **4**, crystallographic mirror planes m and tin-tin distances [Å] marked in violet, distance [Å] of the iodine atom above the basal plane in square brackets; symmetry operations used to generate equivalent atoms: ^1^) x, y-1, z; ^2^) x-1/2, y-1, -z+1/2; ^3^) x-1/2, y, -z+1/2.


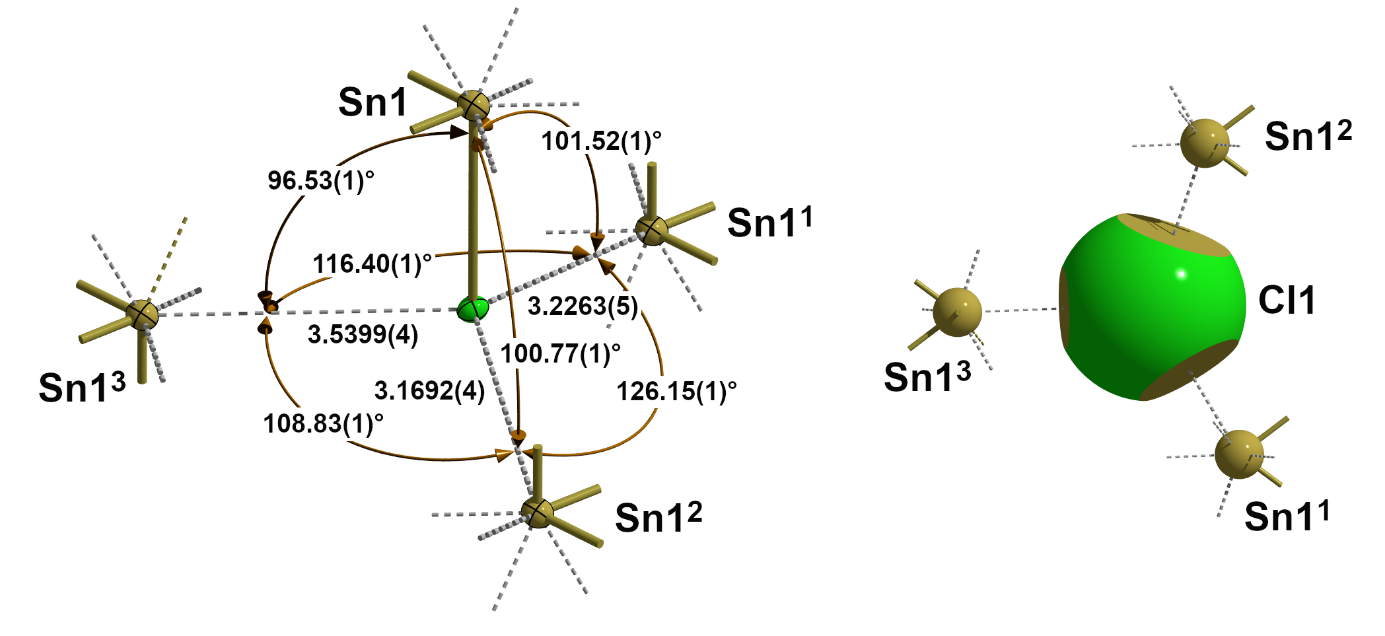


Figure S10: Ball-and-stick model (**left**) and space-filling model (**right**) with interatomic distances and angles describing the distorted tetrahedral tin environment of the chlorine atom in **5**; symmetry operations used to generate equivalent atoms: ^1^) 1-x, -y, 1-z; ^2^) 1-x,1-y, 1-z; ^3^) 1+x, y, z.


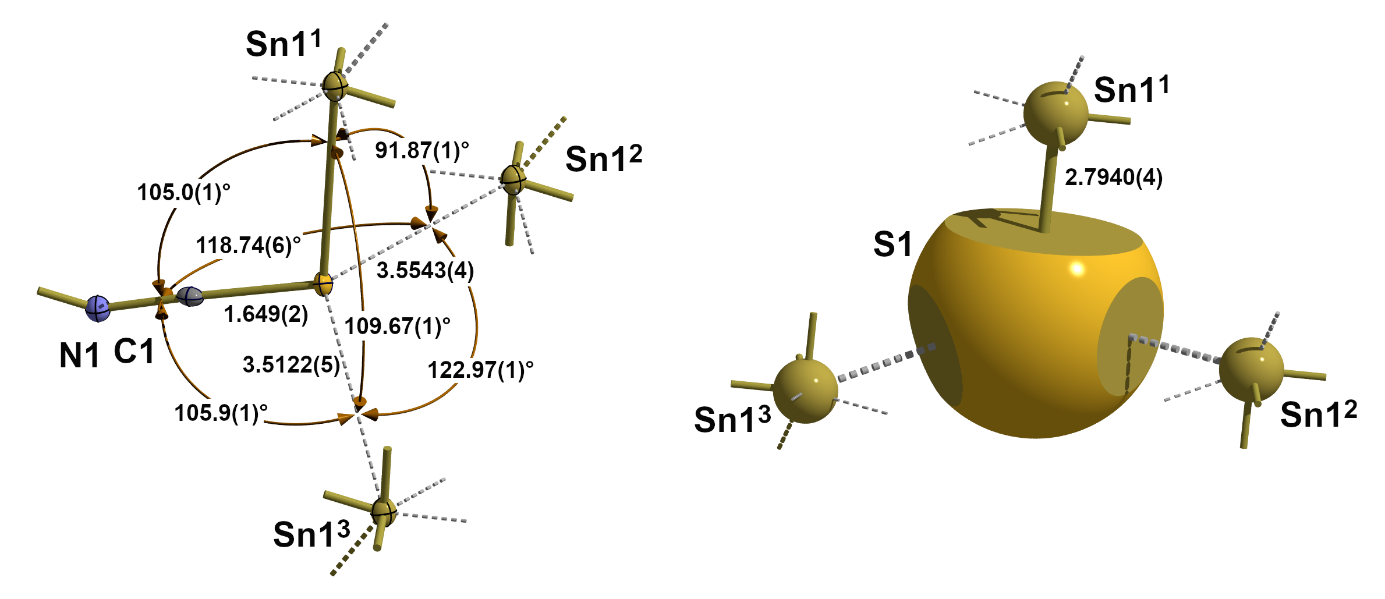


Figure S11: Ball-and-stick model (**left**) and space-filling model (**right**) with interatomic distances [Å] and angles describing the distorted tetrahedral tin environment of the sulphur atom in **5**; symmetry operations used to generate equivalent atoms: ^1^) x,1+y, z; ^2^) 1+x, 1+y, z; ^3^) 1-x, 1-y, 2-z.

## Tables

Table S1: Atomic coordinates ( x 10^4^) and equivalent isotropic displacement parameters (Å^2^ x 10^3^) for SnF(NCS). U(eq) is defined as one third of the trace of the orthogonalized Uij tensor.

|  | x | y | z | U(eq) |
| --- | --- | --- | --- | --- |
| Sn(1) | 1314(1) | 2500 | 3977(1) | 10(1) |
| F(1) | -784(2) | 2500 | 5269(1) | 12(1) |
| S(1) | 5854(1) | 2500 | 7022(1) | 13(1) |
| N(1) | 3180(3) | 2500 | 5414(2) | 16(1) |
| C(1) | 4305(3) | 2500 | 6066(2) | 12(1) |

Table S2: Bond lengths [Å] and angles [°] for SnF(NCS).

| Bond lengths |  |  | Bond angles |  |
| --- | --- | --- | --- | --- |
| Sn(1)-F(1) | 2.218(1) |  | F(1)- Sn(1)-N(1) | 85.44(6) |
| Sn(1)-N(1) | 2.226(2) |  | F(1)- Sn(1)-F(1)#1 | 67.23(3) |
| Sn(1)-F(1)#1 | 2.3672(5) |  | N(1)- Sn(1)-F(1)#1 | 79.31(4) |
| Sn(1)-F(1)#2 | 2.3672(5) |  | F(1)- Sn(1)-F(1)#2 | 67.23(3) |
| Sn(1)-S(1)#3 | 3.2681(4) |  | N(1)- Sn(1)-F(1)#2 | 79.31(4) |
| Sn(1)-S(1)#4 | 3.2681(4) |  | F(1)#1-Sn(1)-F(1)#2 | 130.71(6) |
| Sn(1)-S(1)#5 | 3.5763(5) |  | F(1)- Sn(1)-S(1)#3 | 136.63(1) |
| Sn(1)-S(1)#6 | 3.5763(5) |  | N(1)- Sn(1)-S(1)#3 | 82.12(4) |
|  |  |  | F(1)#1-Sn(1)-S(1)#3 | 69.67(3) |
|  |  |  | F(1)#2-Sn(1)-S(1)#3 | 148.14(3) |
|  |  |  | F(1)- Sn(1)-S(1)#4 | 136.63(1) |
|  |  |  | N(1)- Sn(1)-S(1)#4 | 82.12(4) |
|  |  |  | F(1)#1-Sn(1)-S(1)#4 | 148.14(3) |
|  |  |  | F(1)#2-Sn(1)-S(1)#4 | 69.67(3) |
|  |  |  | S(1)#3-Sn(1)-S(1)#4 | 82.35(1) |
|  |  |  | F(1)- Sn(1)-S(1)#5 | 97.10(3) |
|  |  |  | N(1)- Sn(1)-S(1)#5 | 142.80(1) |
|  |  |  | F(1)#1-Sn(1)-S(1)#5 | 135.82(3) |
|  |  |  | F(1)#2-Sn(1)-S(1)#5 | 67.88(3) |
|  |  |  | S(1)#3-Sn(1)-S(1)#5 | 117.42(1) |
|  |  |  | S(1)#4-Sn(1)-S(1)#5 | 70.63(1) |
|  |  |  | F(1)- Sn(1)-S(1)#6 | 97.10(3) |
|  |  |  | N(1)- Sn(1)-S(1)#6 | 142.80(1) |
|  |  |  | F(1)#1-Sn(1)-S(1)#6 | 67.88(3) |
|  |  |  | F(1)#2-Sn(1)-S(1)#6 | 135.82(3) |
|  |  |  | S(1)#3-Sn(1)-S(1)#6 | 70.63(1) |
|  |  |  | S(1)#4-Sn(1)-S(1)#6 | 117.42(1) |
|  |  |  | S(1)#5-Sn(1)-S(1)#6 | 73.97(1) |
|  |  |  |  |  |
| F(1)-Sn(1) | 2.218(1) |  | Sn(1)- F(1)-Sn(1)#1 | 112.77(3) |
| F(1)-Sn(1)#1 | 2.3672(5 |  | Sn(1)- F(1)-Sn(1)#2 | 112.77(3) |
| F(1)-Sn(1)#2 | 2.3672(5) |  | Sn(1)#1-F(1)-Sn(1)#2 | 130.71(6) |
|  |  |  |  |  |
| S(1)-C(1) | 1.640(2) |  | C(1)- S(1)-Sn(1)#3 | 102.55(6) |
| S(1)-Sn(1)#3 | 3.2680(4) |  | C(1)- S(1)-Sn(1)#4 | 102.55(6) |
| S(1)-Sn(1)#4 | 3.2680(4) |  | Sn(1)#3-S(1)-Sn(1)#4 | 82.35(1) |
| S(1)-Sn(1)#7 | 3.5763(5) |  | C(1)- S(1)-Sn(1)#7 | 97.18(6) |
| S(1)-Sn(1)#8 | 3.5763(5) |  | Sn(1)#3-S(1)-Sn(1)#7 | 98.36(1) |
|  |  |  | Sn(1)#4-S(1)-Sn(1)#7 | 159.63(2) |
|  |  |  | C(1)- S(1)-Sn(1)#8 | 97.18(6) |
|  |  |  | Sn(1)#3-S(1)-Sn(1)#8 | 159.63(2) |
|  |  |  | Sn(1)#7-S(1)-Sn(1)#8 | 73.97(1) |
|  |  |  |  |  |
| N(1)-C(1) | 1.156(3) |  | C(1)-N(1)-Sn(1) | 171.9(2) |
| C(1)-S(1) | 1.640(2) |  | N(1)-C(1)-S(1) | 178.1(2) |

Symmetry transformations used to generate equivalent atoms: #1 -x,-y+1,-z+1 #2 -x,-y,-z+1 #3 -x+1,-y+1,-z+1 #4 -x+1,-y,-z+1 #5 -x+1/2,-y,z-1/2 #6 -x+1/2,-y,z-1/2, #7 -x+1/2,-y+1,z+1/2 #8 -x+1/2,-y,z+1/2

Table S3: Anisotropic displacement parameters (Å^2^ x 10^3^) for SnF(NCS). The anisotropic displacement factor exponent takes the form: -2 πi^2^ [ h^2^ a*^2^ U11 + ... + 2 h k a* b* U12].

|  | U11 | U22 | U33 | U23 | U13 | U12 |
| --- | --- | --- | --- | --- | --- | --- |
| Sn(1) | 9(1) | 12(1) | 10(1) | 0 | 0(1) | 0 |
| F(1) | 13(1) | 7(1) | 17(1) | 0 | 4(1) | 0 |
| S(1) | 11(1) | 16(1) | 12(1) | 0 | -1(1) | 0 |
| N(1) | 15(1) | 18(1) | 17(1) | 0 | -2(1) | 0 |
| C(1) | 12(1) | 9(1) | 13(1) | 0 | 4(1) | 0 |

Table S4: Atomic coordinates ( x 10^4^) and equivalent isotropic displacement parameters (Å^2^ x 10^3^) for SnCl(NCS). U(eq) is defined as one third of the trace of the orthogonalized Uij tensor.

|  | x | y | z | U(eq) |
| --- | --- | --- | --- | --- |
| Sn(1) | 7624(1) | 7500 | 3294(1) | 14(1) |
| S(1) | 1826(1) | 7500 | 5110(1) | 12(1) |
| C(1) | 3688(2) | 7500 | 4471(1) | 11(1) |
| N(1) | 4931(2) | 7500 | 3992(1) | 15(1) |
| Cl(1) | 6119(1) | 7500 | 2204(1) | 15(1) |

Table S5: Bond lengths [Å] and angles [°] for SnCl(NCS).

| Bond lengths |  |  | Bond angles |  |
| --- | --- | --- | --- | --- |
| Sn(1)-N(1) | 2.261(2) |  | N(1)- Sn(1)-Cl(1) | 80.54(3) |
| Sn(1)-Cl(1) | 2.7756(3) |  | N(1)- Sn(1)-Cl(1)#1 | 80.54(3) |
| Sn(1)-Cl(1)#1 | 2.7756(3) |  | Cl(1)- Sn(1)-Cl(1)#1 | 96.30(1) |
| Sn(1)-S(1)#2 | 2.9915(4) |  | N(1)- Sn(1)-S(1)#2 | 80.66(3) |
| Sn(1)-S(1)#3 | 2.9915(4) |  | Cl(1)- Sn(1)-S(1)#2 | 160.66(1) |
| Sn(1)-Cl(1)#4 | 3.4460(4) |  | Cl(1)#1-Sn(1)-S(1)#2 | 85.06(1) |
| Sn(1)-Cl(1)#5 | 3.4460(4) |  | N(1)- Sn(1)-S(1)#3 | 80.66(3) |
|  |  |  | Cl(1)- Sn(1)-S(1)#3 | 85.06(1) |
|  |  |  | Cl(1)#1-Sn(1)-S(1)#3 | 160.66(1) |
|  |  |  | S(1)#2- Sn(1)-S(1)#3 | 87.44(1) |
|  |  |  | N(1)- Sn(1)-Cl(1)#4 | 141.91(1) |
|  |  |  | Cl(1)- Sn(1)-Cl(1)#4 | 131.99(1) |
|  |  |  | Cl(1)#1-Sn(1)-Cl(1)#4 | 77.01(1) |
|  |  |  | S(1)#2- Sn(1)-Cl(1)#4 | 67.17(1) |
|  |  |  | S(1)#3- Sn(1)-Cl(1)#4 | 116.19(1) |
|  |  |  | N(1)- Sn(1)-Cl(1)#5 | 141.91(1) |
|  |  |  | Cl(1)- Sn(1)-Cl(1)#5 | 77.01(1) |
|  |  |  | Cl(1)#1-Sn(1)-Cl(1)#5 | 131.99(1) |
|  |  |  | S(1)#2- Sn(1)-Cl(1)#5 | 116.19(1) |
|  |  |  | S(1)#3- Sn(1)-Cl(1)#5 | 67.17(1) |
|  |  |  | Cl(1)#4-Sn(1)-Cl(1)#5 | 73.74(1) |
|  |  |  |  |  |
| S(1)-C(1) | 1.647(2) |  | C(1)- S(1)-Sn(1)#2 | 104.19(5) |
| S(1)-Sn(1)#2 | 2.9915(4) |  | C1)- S(1)-Sn(1)#3 | 104.19(5) |
| S(1)-Sn(1)#3 | 2.9915(4) |  | Sn(1)#2-S(1)-Sn(1)#3 | 87.44(1) |
|  |  |  |  |  |
| Cl(1)-Sn(1) | 2.7756(3) |  | Sn(1)- Cl(1)-Sn(1)#6 | 96.30(1) |
| Cl(1)-Sn(1)#6 | 2.7756(3) |  | Sn(1)- Cl(1)-Sn(1)#7 | 150.41(2) |
| Cl(1)-Sn(1)#7 | 3.4460(4) |  | Sn(1)#6-Cl(1)-Sn(1)#7 | 88.61(1) |
| Cl(1)-Sn(1)#8 | 3.4460(4) |  | Sn(1)- Cl(1)-Sn(1)#8 | 88.61(1) |
|  |  |  | Sn(1)#6-Cl(1)-Sn(1)#8 | 150.41(2) |
|  |  |  | Sn(1)#7-Cl(1)-Sn(1)#8 | 73.74(1) |
|  |  |  |  |  |
| N(1)-C(1) | 1.158(2) |  | C(1)-N(1)-Sn(1) | 170.9(2) |
| C(1)-S(1) | 1.647(2) |  | N(1)-C(1)-S(1) | 177.7(2) |

*Symmetry transformations used to generate equivalent atoms: #1 x,y+1,z #2 -x+1,y+1/2,-z+1 #3 -x+1,y-1/2,-z+1 #4 x+1/2,y+1,-z+1/2 #5 x+1/2,y,-z+1/2 #6 x,y-1,z #7 x-1/2,y-1,-z+1/2 #8 x-1/2,y,-z+1/2*

Table S6: Anisotropic displacement parameters (Å^2^ x 10^3^) for SnCl(NCS). The anisotropic displacement factor exponent takes the form: -2 πi^2^ [ h^2^ a*^2^ U11 + ... + 2 h k a* b* U12].

|  | U11 | U22 | U33 | U23 | U13 | U12 |
| --- | --- | --- | --- | --- | --- | --- |
| Sn(1) | 10(1) | 19(1) | 12(1) | 0 | 3(1) | 0 |
| S(1) | 9(1) | 15(1) | 12(1) | 0 | 2(1) | 0 |
| C(1) | 13(1) | 10(1) | 11(1) | 0 | -3(1) | 0 |
| N(1) | 13(1) | 17(1) | 16(1) | 0 | 2(1) | 0 |
| Cl(1) | 15(1) | 15(1) | 14(1) | 0 | -2(1) | 0 |

Table S7: Atomic coordinates ( x 10^4^) and equivalent isotropic displacement parameters (Å^2^ x 10^3^) for SnBr(NCS). U(eq) is defined as one third of the trace of the orthogonalized Uij tensor.

|  | x | y | z | U(eq) |
| --- | --- | --- | --- | --- |
| Sn(1) | 7647(1) | 7500 | 3366(1) | 12(1) |
| S(1) | 1860(1) | 7500 | 5109(1) | 9(1) |
| C(1) | 3691(4) | 7500 | 4486(2) | 12(1) |
| N(1) | 4947(3) | 7500 | 4026(2) | 15(1) |
| Br(1) | 6141(1) | 7500 | 2167(1) | 11(1) |

Table S8: Bond lengths [Å] and angles [°] for SnBr(NCS).

| Bond lengths |  |  | Bond angles |  |
| --- | --- | --- | --- | --- |
| Sn(1)-N(1) | 2.271(2) |  | N(1)- Sn(1)-Br(1) | 81.66(4) |
| Sn(1)-Br(1) | 2.9122(2) |  | N(1)- Sn(1)-Br(1)#1 | 81.66(4) |
| Sn(1)-Br(1)#1 | 2.9122(2) |  | Br(1)- Sn(1)-Br(1)#1 | 93.07(1) |
| Sn(1)-S(1)#2 | 2.9881(5) |  | N(1)- Sn(1)-S(1)#2 | 80.90(5) |
| Sn(1)-S(1)#3 | 2.9881(5) |  | Br(1)- Sn(1)-S(1)#2 | 162.51(1) |
| Sn(1)-Br(1)#4 | 3.5037(3) |  | Br(1)#1-Sn(1)-S(1)#2 | 85.81(1) |
| Sn(1)-Br(1)#5 | 3.5037(3) |  | N(1)- Sn(1)-S(1)#3 | 80.90(5) |
|  |  |  | Br(1)- Sn(1)-S(1)#3 | 85.81(1) |
|  |  |  | Br(1)#1-Sn(1)-S(1)#3 | 162.51(2) |
|  |  |  | S(1)#2- Sn(1)-S(1)#3 | 90.04(2) |
|  |  |  | N(1)- Sn(1)-Br(1)#4 | 142.12(1) |
|  |  |  | Br(1)- Sn(1)-Br(1)#4 | 129.01(1) |
|  |  |  | Br(1)#1-Sn(1)-Br(1)#4 | 75.75(1) |
|  |  |  | S(1)#2- Sn(1)-Br(1)#4 | 67.61(1) |
|  |  |  | S(1)#3- Sn(1)-Br(1)#4 | 118.19(1) |
|  |  |  | N(1)- Sn(1)-Br(1)#5 | 142.12(1) |
|  |  |  | Br(1)- Sn(1)-Br(1)#5 | 75.75(1) |
|  |  |  | Br(1)#1-Sn(1)-Br(1)#5 | 129.01(1) |
|  |  |  | S(1)#2- Sn(1)-Br(1)#5 | 118.19(1) |
|  |  |  | S(1)#3- Sn(1)-Br(1)#5 | 67.61(1) |
|  |  |  | Br(1)#4-Sn(1)-Br(1)#5 | 74.21(1) |
|  |  |  |  |  |
| S(1)-C(1) | 1.649(3) |  | C(1)- S(1)-Sn(1)#2 | 104.38(7) |
| S(1)-Sn(1)#2 | 2.9881(5) |  | C1)- S(1)-Sn(1)#3 | 104.38(7) |
| S(1)-Sn(1)#3 | 2.9881(5) |  | Sn(1)#2-S(1)-Sn(1)#3 | 90.04(2) |
|  |  |  |  |  |
| Br(1)-Sn(1) | 2.7756(3) |  | Sn(1)- Br(1)-Sn(1)#6 | 93.07(1) |
| Br(1)-Sn(1)#6 | 2.7756(3) |  | Sn(1)- Br(1)-Sn(1)#7 | 149.47(1) |
| Br(1)-Sn(1)#7 | 3.4460(4) |  | Sn(1)#6-Br(1)-Sn(1)#7 | 89.18(1) |
| Br(1)-Sn(1)#8 | 3.4460(4) |  | Sn(1)- Br(1)-Sn(1)#8 | 89.18(1) |
|  |  |  | Sn(1)#6-Br(1)-Sn(1)#8 | 149.47(1) |
|  |  |  | Sn(1)#7-Br(1)-Sn(1)#8 | 74.21(1) |
|  |  |  |  |  |
| N(1)-C(1) | 1.158(2) |  | C(1)-N(1)-Sn(1) | 170.9(2) |
| C(1)-S(1) | 1.647(2) |  | N(1)-C(1)-S(1) | 177.7(2) |

*Symmetry transformations used to generate equivalent atoms: #1 x,y+1,z #2 -x+1,y+1/2,-z+1 #3 -x+1,y-1/2,-z+1 #4 x+1/2,y+1,-z+1/2 #5 x+1/2,y,-z+1/2 #6 x,y-1,z #7 x-1/2,y-1,-z+1/2 #8 x-1/2,y,-z+1/2*

Table S9: Anisotropic displacement parameters (Å^2^ x 10^3^) for SnBr(NCS). The anisotropic displacement factor exponent takes the form: -2 πi^2^ [ h^2^ a*^2^ U11 + ... + 2 h k a* b* U12].

|  | U11 | U22 | U33 | U23 | U13 | U12 |
| --- | --- | --- | --- | --- | --- | --- |
| Sn(1) | 9(1) | 14(1) | 12(1) | 0 | 2(1) | 0 |
| S(1) | 7(1) | 12(1) | 9(1) | 0 | 2(1) | 0 |
| C(1) | 14(1) | 11(1) | 10(1) | 0 | -2(1) | 0 |
| N(1) | 14(1) | 15(1) | 15(1) | 0 | 2(1) | 0 |
| Br(1) | 11(1) | 10(1) | 11(1) | 0 | -2(1) | 0 |

Table S10: Atomic coordinates ( x 10^4^) and equivalent isotropic displacement parameters (Å^2^ x 10^3^) for SnI(NCS). U(eq) is defined as one third of the trace of the orthogonalized Uij tensor

|  | x | y | z | U(eq) |
| --- | --- | --- | --- | --- |
| Sn(1) | 7543(1) | 7500 | 3438(1) | 12(1) |
| S(1) | 1940(1) | 7500 | 5120(1) | 11(1) |
| C(1) | 3719(5) | 7500 | 4518(3) | 9(1) |
| N(1) | 4939(5) | 7500 | 4076(3) | 14(1) |
| I(1) | 6089(1) | 7500 | 2122(1) | 12(1) |

Table S11: Bond lengths [Å] and angles [°] for SnI(NCS).

| Bond lengths |  |  | Bond angles |  |
| --- | --- | --- | --- | --- |
| Sn(1)-N(1) | 2.261(4) |  | N(1)- Sn(1)-S(1)#1 | 81.61(7) |
| Sn(1)-S(1)#1 | 3.0093(7) |  | N(1)- Sn(1)-S(1)#2 | 81.61(7) |
| Sn(1)-S(1)#2 | 3.0093(7) |  | S(1)#1- Sn(1)-S(1)#2 | 92.43(3) |
| Sn(1)-I(1) | 2.9881(5) |  | N(1)- Sn(1)-I(1) | 84.10(7) |
| Sn(1)-I(1)#3 | 2.9881(5) |  | S(1)#1- Sn(1)-I(1) | 165.60(2) |
| Sn(1)-I(1)#4 | 3.0865(3) |  | S(1)#2- Sn(1)-I(1) | 87.27(2) |
| Sn(1)-I(1)#5 | 3.0865(3) |  | N(1)- Sn(1)-I(1)#3 | 84.10(7) |
|  |  |  | S(1)#1- Sn(1)-I(1)#3 | 87.27(2) |
|  |  |  | S(1)#2- Sn(1)-I(1)#3 | 165.60(2) |
|  |  |  | I(1) - Sn(1)-I(1)#3 | 89.48(1) |
|  |  |  | N(1)- Sn(1)-I(1)#4 | 142.73(2) |
|  |  |  | S(1)#1- Sn(1)-I(1)#4 | 67.15(2) |
|  |  |  | S(1)#2- Sn(1)-I(1)#4 | 118.13(2) |
|  |  |  | I(1)- Sn(1)-I(1)#4 | 125.34(1) |
|  |  |  | I(1)#3- Sn(1)-I(1)#4 | 74.93(1) |
|  |  |  | N(1)- Sn(1)-I(1)#5 | 142.73(2) |
|  |  |  | S(1)#1- Sn(1)-I(1)#5 | 118.13(2) |
|  |  |  | S(1)#2- Sn(1)-I(1)#5 | 67.15(2) |
|  |  |  | I(1)- Sn(1)-I(1)#5 | 74.93(1) |
|  |  |  | I(1)#3- Sn(1)-I(1)#5 | 125.34(1) |
|  |  |  | I(1)#4- Sn(1)-I(1)#5 | 73.11(1) |
|  |  |  |  |  |
| S(1)-C(1) | 1.652(4) |  | C(1)- S(1)-Sn(1)#1 | 103.4(1) |
| S(1)-Sn(1)#1 | 3.0093(7) |  | C1)- S(1)-Sn(1)#2 | 103.4(1) |
| S(1)-Sn(1)#2 | 3.0093(7) |  | Sn(1)#1-S(1)-Sn(1)#2 | 92.43(3) |
|  |  |  |  |  |
| I(1)-Sn(1) | 3.0865(3) |  | Sn(1)- I(1)-Sn(1)#6 | 89.48(1) |
| I(1)-Sn(1)#6 | 3.0865(3) |  | Sn(1)- I(1)-Sn(1)#7 | 147.17(1) |
| I(1)-Sn(1)#7 | 3.6478(4) |  | Sn(1)#6-I(1)-Sn(1)#7 | 90.11(1) |
| I(1)-Sn(1)#8 | 3.6478(4) |  | Sn(1)- I(1)-Sn(1)#8 | 90.11(1) |
|  |  |  | Sn(1)#6-I(1)-Sn(1)#8 | 147.17(1) |
|  |  |  | Sn(1)#7-I(1)-Sn(1)#8 | 73.10(1) |
|  |  |  |  |  |
| N(1)-C(1) | 1.155(5) |  | C(1)-N(1)-Sn(1) | 170.8(4) |
| C(1)-S(1) | 1.652(4) |  | N(1)-C(1)-S(1) | 178.2(4) |

*Symmetry transformations used to generate equivalent atoms: #1 -x+1,y+1/2,-z+1 #2 -x+1,y-1/2,-z+1 #3 x,y+1,z #4 x+1/2,y+1,-z+1/2 #5 x+1/2,y,-z+1/2 #6 x,y-1,z #7 x-1/2,y-1,-z+1/2 #8 x-1/2,y,-z+1/2*

Table S12: Anisotropic displacement parameters (Å^2^ x 10^3^) for SnI(NCS). The anisotropic displacement factor exponent takes the form: -2 πi^2^ [ h^2^ a*^2^ U11 + ... + 2 h k a* b* U12].

|  | U11 | U22 | U33 | U23 | U13 | U12 |
| --- | --- | --- | --- | --- | --- | --- |
| Sn(1) | 10(1) | 13(1) | 12(1) | 0 | 2(1) | 0 |
| S(1) | 8(1) | 14(1) | 10(1) | 0 | 2(1) | 0 |
| C(1) | 12(2) | 9(2) | 8(2) | 0 | 0(1) | 0 |
| N(1) | 12(2) | 16(2) | 15(2) | 0 | 3(1) | 0 |
| I(1) | 11(1) | 11(1) | 13(1) | 0 | -2(1) | 0 |

Table S13: Atomic coordinates ( x 10^4^) and equivalent isotropic displacement parameters (Å^2^ x 10^3^) for β-SnCl(NCS). U(eq) is defined as one third of the trace of the orthogonalized Uij tensor.

|  |  | y | z | U(eq) |
| --- | --- | --- | --- | --- |
| Sn(1) | 3855(1) | 1960(1) | 6990(1) | 11(1) |
| Cl(1) | 7451(1) | 2531(1) | 4614(1) | 12(1) |
| S(1) | 7867(5) | 8692(1) | 8860(3) | 11(1) |
| N(1) | 6949(3) | 4205(3) | 8123(2) | 15(1) |
| C(1) | 7273(3 | 6083(3) | 8411(2) | 11(1) |

Table S14: Bond lengths [Å] and angles [°] for β-SnCl(NCS).

| Bond lengths |  |  | Bond angles |  |
| --- | --- | --- | --- | --- |
| Sn(1)-N(1) | 2.305(1) |  | N(1)- Sn(1)-Cl(1) | 85.26(4) |
| Sn(1)-Cl(1) | 2.5501(4) |  | N(1)- Sn(1)-S(1)#1 | 73.56(4) |
| Sn(1)-S(1)#1 | 2.7940(4) |  | Cl(1)- Sn(1)-S(1)#1 | 89.43(1) |
| Sn(1)-Cl(1)#2 | 3.1692(4) |  | N(1)- Sn(1)-Cl(1)#2 | 71.56(4) |
| Sn(1)-Cl(1)#3 | 3.2263(4) |  | Cl(1)- Sn(1)-Cl(1)#2 | 79.23(1) |
| Sn(1)-S(1)#4 | 3.5122(4) |  | S(1)#1- Sn(1)-Cl(1)#2 | 144.02(1) |
| Sn(1)-Cl(1)#5 | 3.5399(4) |  | N(1)- Sn(1)-Cl(1)#3 | 152.11(4) |
|  |  |  | Cl(1)- Sn(1)-Cl(1)#3 | 78.48(1) |
|  |  |  | S(1)#1- Sn(1)-Cl(1)#3 | 83.69(1) |
|  |  |  | Cl(1)#2-Sn(1)-Cl(1)#3 | 126.15(1) |
|  |  |  | N(1)- Sn(1)-S(1)#4 | 71.29(4) |
|  |  |  | Cl(1)- Sn(1)-S(1)#4 | 152.34(1) |
|  |  |  | S(1)#1- Sn(1)-S(1)#4 | 70.33(1) |
|  |  |  | Cl(1)#2-Sn(1)-S(1)#4 | 105.89(1) |
|  |  |  | Cl(1)#3-Sn(1)-S(1)#4 | 116.22(1) |
|  |  |  | N(1)- Sn(1)-Cl(1)#5 | 141.62(4) |
|  |  |  | Cl(1)- Sn(1)-Cl(1)#5 | 96.53(1) |
|  |  |  | S(1)#1- Sn(1)-Cl(1)#5 | 144.57(1) |
|  |  |  | Cl(1)#2-Sn(1)-Cl(1)#5 | 71.17(1) |
|  |  |  | Cl(1)#3-Sn(1)-Cl(1)#5 | 63.60(1) |
|  |  |  | S(1)#4- Sn(1)-Cl(1)#5 | 110.93(1) |
|  |  |  |  |  |
| Cl(1)-Sn(1)#2 | 3.1692(4) |  | Sn(1)- Cl(1)-Sn(1)#2 | 100.77(1) |
| Cl(1)-Sn(1)#3 | 3.2263(4) |  | Sn(1)- Cl(1)-Sn(1)#3 | 101.52(1) |
| Cl(1)-Sn(1)#6 | 3.5399(4) |  | Sn(1)#2-Cl(1)-Sn(1)#3 | 126.15(1) |
|  |  |  | Sn(1)- Cl(1)-Sn(1)#6 | 96.53(1) |
|  |  |  | Sn(1)#2-Cl(1)-Sn(1)#6 | 108.83(1) |
|  |  |  | Sn(1)#3-Cl(1)-Sn(1)#6 | 116.40(1) |
|  |  |  | Sn(1)- Cl(1)-Sn(1)#7 | 136.46(1) |
|  |  |  | Sn(1)#2-Cl(1)-Sn(1)#7 | 121.18(1) |
|  |  |  | Sn(1)#3-Cl(1)-Sn(1)#7 | 64.18(1) |
|  |  |  | Sn(1)#6-Cl(1)-Sn(1)#7 | 61.14(1) |
|  |  |  |  |  |
| S(1)-C(1)  S(1)-Sn(1)#8  S(1)-Sn(1)#4  S(1)-Sn(1)#9 | 1.649(2)  2.7940(4)  3.5122(4)  3.5543(4) |  | C(1)- S(1)-Sn(1)#8  C(1)- S(1)-Sn(1)#4  Sn(1)#8-S(1)-Sn(1)#4  Sn(1)#8-S(1)-Sn(1)#9  Sn(1)#4-S(1)-Sn(1)#9  Sn(1)#8-S(1)-Sn(1)  Sn(1)#4-S(1)-Sn(1)  Sn(1)#9-S(1)-Sn(1) | 105.0(1)  105.9(1)  109.67(1)  91.87(1)  122.97(1)  91.46(1)  107.80(1)  124.25(1) |
|  |  |  |  |  |
| N(1)-C(1) | 1.165(2) |  | C(1)-N(1)-Sn(1) | 144.8(1) |

*Symmetry transformations used to generate equivalent atoms: #1 x,y-1,z #2 -x+1,-y+1,-z+1 #3 -x+1,-y,-z+1 #4 -x+1,-y+1,-z+2 #5 x-1,y,z #6 x+1,y,z #7 -x+2,-y,-z+1 #8 x,y+1,z #9 x+1,y+1,z*

Table S15: Anisotropic displacement parameters (Å^2^ x 10^3^) for SnI(NCS). The anisotropic displacement factor exponent takes the form: -2 πi^2^ [ h^2^ a*^2^ U11 + ... + 2 h k a* b* U12].

|  | U11 | U22 | U33 | U23 | U13 | U12 |
| --- | --- | --- | --- | --- | --- | --- |
| Sn(1) | 11(1) | 11(1) | 12(1) | -3(1) | 1(1) | -1(1) |
| Cl(1) | 13(1) | 12(1) | 11(1) | -2(1) | 2(1) | 1(1) |
| S1(1) | 14(1) | 8(1) | 11(1) | -3(1) | -1(1) | -1(1) |
| N(1) | 17(2) | 12(1) | 15(1) | -3(1) | -3(1) | -1(1) |
| C(1) | 10(1) | 12(1) | 9(1) | 1(1) | -2(1) | 1(1) |

Table S16: Calculated interpenetration indices p and bond valences v.

| **compound** | **Sn-X** | **d(Sn-X) [Å]** | ***ρ*** | ***v*** |
| --- | --- | --- | --- | --- |
|  |  |  |  |  |
| **SnF(NCS)** |  |  |  |  |
|  | Sn-N | 2.226(2) | 92.22 | 0.61 |
|  | Sn-F | 2.218(1) | 84.64 | 0.45 |
|  | Sn-F | 2.3672(5) | 75.76 | 0.30 |
|  | Sn-S | 3.2681(4) | 45.88 | 0.10 |
|  | Sn-S | 3.5763(5) | 25.73 | 0.04 |
|  |  |  |  |  |
| **α-SnCl(NCS)** |  |  |  |  |
|  | Sn-N | 2.261(2) | 90.06 | 0.56 |
|  | Sn-Cl | 2.7756(3) | 75.79 | 0.30 |
|  | Sn-S | 2.9915(4) | 63.95 | 0.22 |
|  | Sn-Cl | 3.4460(4) | 34.25 | 0.05 |
|  |  |  |  |  |
| **SnBr(NCS)** |  |  |  |  |
|  | Sn-N | 2.271(2) | 89.44 | 0.54 |
|  | Sn-Br | 2.9122(2) | 77.15 | 0.33 |
|  | Sn-S | 2.9881(5) | 64.18 | 0.22 |
|  | Sn-Br | 3.5037(3) | 35.20 | 0.07 |
|  |  |  |  |  |
| **SnI(NCS)** |  |  |  |  |
|  | Sn-N | 2.261(4) | 90.06 | 0.56 |
|  | Sn-I | 3.0865(3) | 77.63 | 0.40 |
|  | Sn-S | 3.0093(7) | 62.79 | 0.21 |
|  | Sn-I | 3.6478(3) | 36.66 | 0.09 |
|  |  |  |  |  |
| **β-SnCl(NCS)** |  |  |  |  |
|  | Sn-Cl | 2.5501(4) | 90.73 | 0.55 |
|  | Sn-N | 2.305(1) | 87.35 | 0.50 |
|  | Sn-S | 2.7940(4) | 76.86 | 0.37 |
|  | Sn-Cl | 3.1692(4) | 50.28 | 0.10 |
|  | Sn-Cl | 3.2263(4) | 45.94 | 0.09 |
|  | Sn-S | 3.5122(4) | 29.92 | 0.05 |
|  | Sn-Cl | 3.5399(4) | 25.17 | 0.04 |
|  | Sn-S | 3.5543(4) | 27.17 | 0.05 |
